# Management Strategy Evaluation Applied to Coral Reef Ecosystems in Support of Ecosystem-Based Management

**DOI:** 10.1371/journal.pone.0152577

**Published:** 2016-03-29

**Authors:** Mariska Weijerman, Elizabeth A. Fulton, Russell E. Brainard

**Affiliations:** 1 Joint Institute for Marine and Atmospheric Research, University of Hawaii at Manoa, Honolulu, Hawaii, United States of America; 2 Environmental Systems Analysis Group, Wageningen University, Wageningen, the Netherlands; 3 Coral Reef Ecosystem Program, Pacific Islands Fisheries Science Center, NOAA Fisheries, Honolulu, Hawaii, United States of America; 4 CSIRO Oceans and Atmosphere, Hobart, Tasmania, Australia; 5 Centre for Marine Socioecology, University of Tasmania, Hobart, Tasmania, Australia; National Taiwan Ocean University, TAIWAN

## Abstract

Ecosystem modelling is increasingly used to explore ecosystem-level effects of changing environmental conditions and management actions. For coral reefs there has been increasing interest in recent decades in the use of ecosystem models for evaluating the effects of fishing and the efficacy of marine protected areas. However, ecosystem models that integrate physical forcings, biogeochemical and ecological dynamics, and human induced perturbations are still underdeveloped. We applied an ecosystem model (Atlantis) to the coral reef ecosystem of Guam using a suite of management scenarios prioritized in consultation with local resource managers to review the effects of each scenario on performance measures related to the ecosystem, the reef-fish fishery (e.g., fish landings) and coral habitat. Comparing tradeoffs across the selected scenarios showed that each scenario performed best for at least one of the selected performance indicators. The integrated ‘full regulation’ scenario outperformed other scenarios with four out of the six performance metrics at the cost of reef-fish landings. This model application quantifies the socio-ecological costs and benefits of alternative management scenarios. When the effects of climate change were taken into account, several scenarios performed equally well, but none prevented a collapse in coral biomass over the next few decades assuming a business-as-usual greenhouse gas emissions scenario.

## Introduction

Sustainable use of environmental resources is inextricably linked to long-term human well-being [1]. This is especially true for many tropical countries and island territories where coral reefs provide provisional and regulatory services to millions of people who depend on them [2]. However, the overwhelming evidence of anthropogenic loss or degradation of coral reef ecosystems worldwide [3, 4] leads to challenges for coral reef managers who need to sustain the ecosystem functions and services under changing environmental conditions and human use patterns [5, 6]. Management decisions intended to achieve desired outcomes have cultural, social and economic consequences for the resource users and these consequences should be accounted for in policy decisions to increase compliance [7, 8]. For example, coral reef fisheries not only provide food but also recreation and cultural identity for local residents [9]. Loss of fishery yield due to over-exploitation, degraded habitats or policy regulations have far-reaching social, cultural and economic consequences for the people relying on these resources. This recognition that humans are an integrated part of the ecosystems, has led a movement toward ecosystem-based management (EBM) in the last decade [10, 11]. Ecosystem models can be useful tools in the planning and implementation of EBM by evaluating ecological and socioeconomic tradeoffs of alternative management policies prior to their implementation [12, 13].

In the last 30 years, research on the efficacy of coral reef fisheries management and implications of alternative management scenarios has seen a steep increase [14]. However, in 80% of these studies conclusions were primarily in general conjectural terms and projections of climate change were often not taken into account [14]. Johnson et al [14] concluded that studies on the effectiveness of different management actions and approaches, tradeoffs and trajectories under climate change are still lacking and this study contributes to filling that gap.

In this paper, we describe the application of an ecosystem model to a complex multispecies fishery with degraded habitats where we evaluate the socio-ecological tradeoffs of alternative management policies, taking into account the projected effects of climate change. We apply the model using the island of Guam in the tropical west Pacific Ocean as a case study. Over the last three decades coral cover and reef-fish biomass have declined in Guam [15]. To enhance conservation of fish stocks and habitat, the government of Guam established five marine protected areas (MPAs) in 1997–2001. MPAs have been shown to be effective at enhancing resilience to climate change [16], increasing coral recovery in the Caribbean [17], supporting larval supplies to other areas [18] and enhancing biomass and reproduction of fish species [19]. The MPAs around Guam have increased fish abundance and reproduction of some fish species compared to the open access areas [20, 21], but fish biomass is still heavily depleted compared to a situation without humans [22]. Guam managers are now interested in using ecosystem modelling to explore tradeoffs of alternative management approaches [23].

Five potential management scenarios were selected after discussions at two informal meetings in Guam with local managers and other stakeholders (S1 Table is a list of participatory agencies). These simulated scenarios are example scenarios to visualize the utility of the ‘end-to-end’ Guam Atlantis model and to help understand and evaluate the socio-ecological tradeoffs of alternative management policies. Policy performance was measured against indicators for ecosystem services (e.g., reef status, biomass of exploited species, total landings). Simulations were modeled with and without the cumulative effects of climate change. Model results of the selected management policies were then compared relative to each other and these results can be used to evaluate the trade-offs inherent in having both socio-economic and ecological goals.

## Methods

### Atlantis ecosystem model

The Atlantis ecosystem model is a deterministic spatially-explicit dynamic model that couples biophysical processes with human-use dynamics and is used as a policy exploration tool for EBM [24]. The main dynamics and process equations are provided in Fulton *et al*. [25] and briefly summarized in S1 Text. In a companion paper [26] we explored the interactive effect size of the three main drivers that influence the reef status in Guam: land-based sources of pollution (LBSP; sediments and nutrients), fishing, and climate change (ocean warming and acidification). In the supplemental materials of that paper we detail the validation of the different stressors by comparing modeled data with empirical data or expected patterns. Modeled effects of LBSP and fishing showed a good overlap with empirical data, although model skill assessment showed that the model was positively biased towards functional species groups with a high biomass, these groups were over-represented (especially the small-bodied parrotfish (scrapers)). Model sensitivity analyses indicated that the LBSP effects, in particular, were sensitive to productivity. Ocean acidification was not well resolved in the model, but the more imminent climate change threat to corals due to ocean warming showed a few bleaching events in the first decade in 2000, after which corals recovered to some extent. These results are in agreement with empirical studies. The model also showed that corals could not keep up when bleaching events were annual, as projected to happen under the current greenhouse gas emission trajectory (IPCC AR8.5). By around 2040 coral biomass had plummeted to very low levels both for branching and massive corals [26]; a result also shown by other studies [27–29]. Despite the limitations of the model (bias, sensitive to productivity, effects of acidification not well resolved), the model was able to project plausible biomass data under the different stressors and was deemed robust for comparative studies [26].

Human-use dynamics can be incorporated in Atlantis through the fishing module, and the management and assessment module. For the purpose of our paper, we are only interested in the relative comparisons of the performance of alternative management scenarios. Fishing was simplified and represented by constant fishing mortality rates (% mortality per year) over the course of the simulation (with variations to represent the management scenarios). We did not include the monitoring and assessment module of the Atlantis model framework. To set our fixed fishing mortality, we first calibrated the model to spatially explicit historical landings of the shore-based fishery and to biomass trends [30, 31]. We calculated the fishing mortality for each functional species group by dividing the landings at year 1 with the standing stock biomass at year 1 to get a proxy for annual fishing mortality for that functional group. We then used those proxy values as the fixed fishing mortality per functional group.

**Fig 1:**
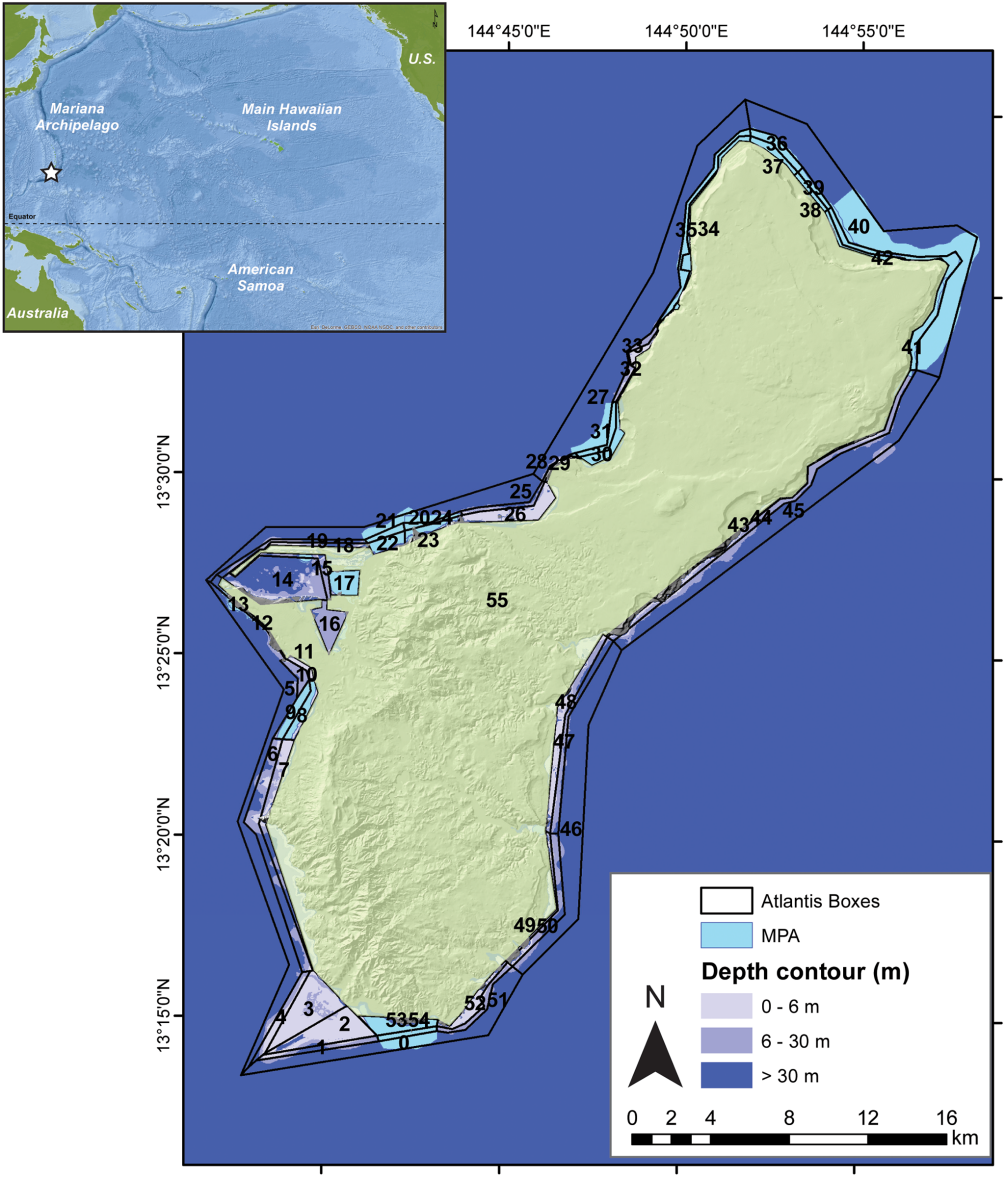
Spatial structure of the Guam Atlantis model based on homogeneous biophysical characteristics. Polygons closest to shore have one depth layer (0–6 m) and the others two (0–6 m and 6–30 m for the middle ones and 0–6 m and 6–100 m for the 7 outer, boundary polygons). Blue polygons indicate marine preserves. The star in the inset map represents the location of Guam located in the Mariana Archipelago in the Pacific Ocean. Polygons with nutrient and/or sediment inputs are those numbered 3, 7, 8, 10, 16, 17, 22, 23, 24, 26, 30, 32, 48, 49, 52, and 53. (Figure taken with permission from [26]).

The recently developed Guam Atlantis model [26] encompasses the shallow (≤ 30 m) reefs around Guam, spanning approximately 110 km^2^ (Fig 1). This model domain incorporates 55 spatially-differentiated habitats (polygons) and 2 vertical water layers (0–6 m and 6–30 m) allowing for the representation of hydrodynamic and biological processes around Guam. These processes are forced with daily hydrodynamic flows, salinity, and temperature outputs from a 5-km resolution, regional ocean modeling system model (ROMS: www.myroms.org) developed for the Coral Triangle region in the western Pacific Ocean [32].

Trophic dynamics are represented by 42 functional groups based on diet, life-history, ecological role, and habitat requirements (S2 Table). Where appropriate, functional groups were further divided into exploited and lightly-fished taxa based on inshore fishery creel surveys conducted by Guam’s Division of Aquatic and Wildlife Resources (DAWR). Initial conditions and data sources are described in Weijerman et al [31] and Weijerman et al [26].

The model was considered robust after passing three calibration tests [33–35] (S1 Text): (1) the model was able to reproduce unfished biomasses, i.e., the model reached and stabilized at similar biomass levels to those observed in marine reserves in Guam and at unpopulated Northern Mariana Islands; (2) weight-at-age stayed stable and abundance of size classes decreased with increasing size classes (few large organisms and many small ones), and (3) the model was able to fit historical catch time series which were derived from DAWR inshore fishery creel surveys [30]. The model was further validated by comparing model output data for key coral reef dynamics (effect of LBSP, mitigating effect of structural complexity on fish predation, coral-algal competition) with empirical data from Guam and published relationships [26] (S1 Text).

### Simulated management scenarios

In November 2012 the Coral Reef Conservation Program management liaison, Ms Valerie Brown, NOAA Pacific Islands Regional Office, facilitated an informal meeting. Invitations to attend an introductory presentation on integrated ecosystem modeling and how it could help managers were sent out to all local and federal managers and other stakeholders (staff of university of Guam, Marine Laboratory, US Navy, NGOs, fisheries Co-op) that are involved with coral reef ecosystems in Guam. Fifteen people representing 11 organizations attended and follow up meetings were organized with staff of organizations that could not attend, reaching out to another 11 people (S1 Table). Based on discussions of management goals and identified management scenarios of interest, we selected six scenarios. In June 2014, we presented those selected scenarios to the resource managers and stakeholders to make sure we captured their interest sufficiently. Apart from those six alternative management scenarios we also simulated a ‘no-change’ or ‘status quo’ scenario and a ‘no stressors’ (no fishing, no LBSP, no climate change) scenario. We identified appropriate ecosystem metrics based on the managers’ goals (S2 Text) and used the model outcome of the no-stressors scenario as the ‘best’ performance criteria, against which we evaluated the ecosystem response of alternative management scenarios of interest (Table 1).

**Table 1:** Goals, ecosystem metrics and performance criteria. Performance of the alternative strategies was based on reaching the criteria of conservation ecosystem metrics (#1–3) and extraction ecosystem metrics (#4a, b). Criteria were based on a simulation of no local (fishing and land-based sources of pollution) or global (climate change) stressors. The criteria for landings are the total catches from the status quo simulation.

Goal	Ecosystem metric	Criteria
		(45 year)
1. Improved water quality	Calcifiers:non-calcifiers ratio	1.15
2a. Increased reef resilience	Biomass of herbivores: browsers & grazers & detritivores	1,344 t
2b. Increased reef resilience	Biomass of herbivores: excavators & scrapers	1,043 t
3. Enhanced fish biomass	Total reef-fish biomass	5,309 t
4a. Maintain or improved fishery landings	Number of fish groups not overexploited	20
4b. Maintain or improved fishery landings	Biomass of reef-fish landings caught by shore-based fishers	128 t

In total, seven policy scenarios were simulated with Guam Atlantis and one ‘no stressors’ scenario. The policy scenarios were:

Scenario 1—Status quo (no change) represented by five MPAs and existing levels of land-based sources of pollution.
  The status-quo simulation had constant fishing mortalities (F) per functional group (S3 Table) with no fishing (F = 0) in the MPAs and additional runoff of LBSP. LBSP was modeled as the addition of nutrients and sediments to the coastal polygons with riverine run-off and/or sewage outfall pipes (Fig 1), and estimated loads were based on flow data and the related sediment and nutrient inputs per river for each Atlantis polygon [31].
Scenario 2—Remove existing MPAs and implement a weekly catch limit (further referred to as TAC) with existing levels of LBSP.
  We estimated an annual catch limit as 75% of the average catches of the first five years of the status quo catches and then converted this value to a weekly TAC. We simulated the TAC scenario with the same fixed fishing mortality rates as in the status quo simulation but when the TAC was reached, fishing stopped for that week. Fishing was allowed in every polygon.
Scenario 3—Remove existing MPAs and implement size limits with existing levels of LBSP.
  For the size-limit-based fishery simulations, we assumed the same fixed fishing mortality rates as in the status quo simulation but no immature fishes were targeted. Since fishing mortality stayed the same under this scenario compared to the status quo scenario, older size classes get targeted more heavily. Based on the weight and age at first maturity, the age and size at first capture for each functional group differed (S3 Table). Fishing was allowed in every polygon.
Scenario 4—Remove existing MPAs and implement TAC and size limits with existing levels of LBSP.
  In this scenario we combined the rules of scenarios 2 and 3.
Scenario 5—Remove existing MPAs and implement TAC and size limits with no additional LBSP.
  This scenario differed only from scenario 4 by not simulating the delivery of additional nutrients and sediments to the coastal areas.
Scenario 6—Status quo with no LBSP.
  In this scenario we used the same constant fishing mortalities as identified under scenario 1, but did not simulate any additional nutrients and sediments to the coastal areas.
Scenario 7—Full regulations. In this scenario we kept existing MPAs in place and implemented both size limits and TAC with no LBSP. This scenario combines all management regulations.
  Each of the scenarios was simulated for 45 years (1985–2030) without including climate change projections. We wanted to take a realistic no-regrets approach to management strategy selection, so the better performing scenarios with no climate change were also re-run for 45 years with climate drivers included. For this approach we selected the three scenarios where the performance evaluation with no climate change had the highest average across all four goals.

### Performance evaluation

Criteria to assess the performance of the scenarios were based on a model simulation with no stressors for ecosystem state conditions and for the fishery the current landings, hence, ecosystem metrics reaching these values were considered ‘best’ (Table 1). Performance of each scenario was measured at the end of the simulation against criteria for six ecosystem metrics based on the four management goals (Table 1 and S2 Text):

1. Improved water quality (no additional LBSP). The metric used to assess the performance of this goal was the ratio of benthic calcifiers to non-calcifiers with calcifiers defined as corals and crustose-coralline algae (CCA) and non-calcifiers as turf and macroalgae.
2. Increased reef resilience. Performance metrics for this goal were biomass of different ecologically important herbivorous fishes, as these groups are critical for maintaining coral-reef habitat and reversing macroalgal strands to cropped states [36].
3. Enhanced fish biomass. Modeled outcome of total fish biomass was used as the metric for the performance of this goal.
4. Maintenance of, or improved fishery landings. Performance for this goal was measured by two fishery-related metrics: (a) the number of functional fish groups that are not overexploited, and (b) landings of reef fish.

To account for interannual variability, we took the mean of the last five simulated years. For ease of interpretation and visualization, the 5-year mean values were normalized over all strategies so that the best result of an ecosystem metric is assigned the value of 1 and all other values scaled accordingly.

Different weightings can be given to the ecosystem metrics in quantifying the overall performance of each management approach. Since managers identified four ecosystem goals and the identified ecosystem metrics are based on those goals, we weighted 1–4 equally and took the average of 2a and 2b for goal 2 and the average of 4a and 4b for goal 4. As the management goals can also be grouped into a conservation component, (goals 1–3) representing the functionality of the ecosystem, and a socio-economic component (goal 4), we also evaluated the cost and benefit tradeoffs between the metrics #1–3 (all weighted equally) and metric #4 by taking the overall average of these two components.

## Results

**Fig 2:**
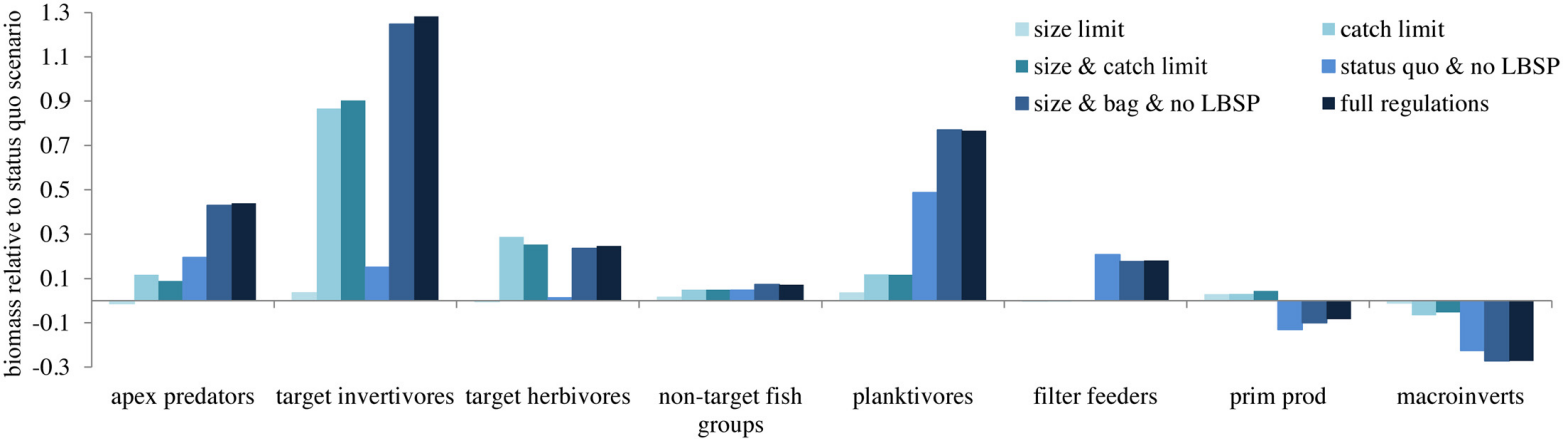
Ecosystem effects after a 45-year simulation of alternative scenarios on different functional groups. Target groups are species exploited in the shore-based fishery. Filter feeders include corals and other sessile benthic filter feeders. Prim prod is primary producers and include phytoplankton and benthic algae.

In general, no one management scenario was best for all goals. However, with regard to fish biomass, as can be expected, fishery regulations that reduce fishing pressure led to a higher biomass of target species (apex predators, herbivores and invertivores), while the non-target groups were less influenced (Fig 2). Surprisingly, the response of target herbivores was less pronounced compared to the target invertivores (but see discussion). Response of invertebrate groups was similar in all fishery regulation and status-quo scenarios (Fig 2). Likely because of an increase in biomass in invertebrate feeders, the overall biomass of invertebrates decreased correspondingly

### No land-based sources of pollution scenarios

Scenarios with no land-based sources of pollution (a range of different fishery regulations) showed a shift in the benthic composition towards an increase in benthic filter feeders (corals, soft corals, sponges, bivalves, anemones, zooanthids), as well as a reduction in the overall biomass of primary producers (benthic algae and phytoplankton), compared to scenarios with LBSP (Fig 2, S1 Fig, S4 Table). Other ecosystem effects included a clear increase in biomass of planktivores (FPL), mid-water piscivores (FPM), rays (RAY), sea stars (BSS) and demersal (ZD) and herbivorous (copepods, ZH) zooplankton and a decrease in the biomass of benthic carnivores (BC), infauna (BM, polychaetes), cephalopods (CEP), and benthic grazers (BG, urchins), (Fig 2, S1 Fig).

### Socio-ecological tradeoffs

**Fig 3:**
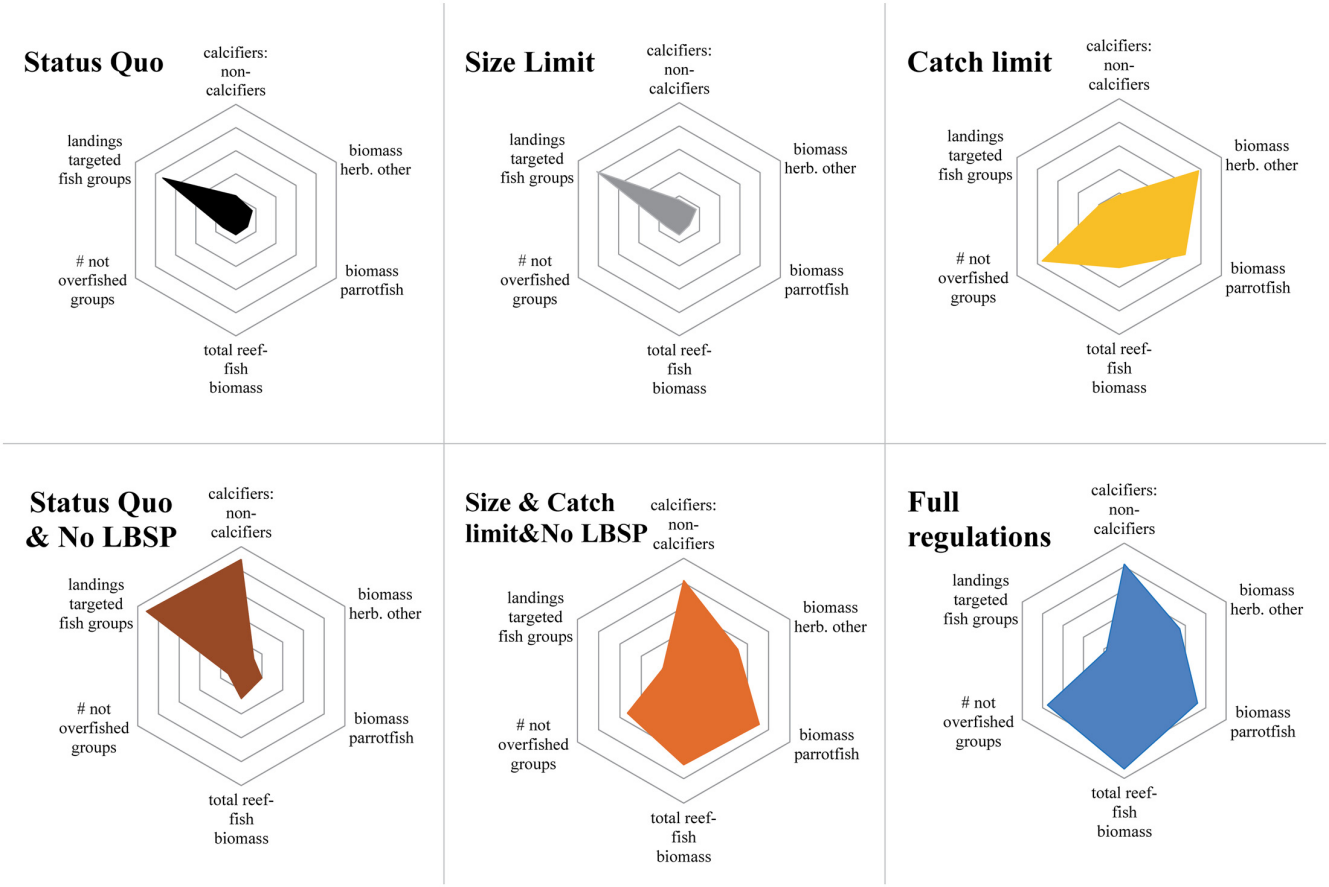
The overall performance of the management strategies for the scaled integrated (composite) performance measures (scaled so that best performance of any scenario in each metric is set to one, represented by the outer circle, and all other responses scaled to that so the larger the area the better the overall ecosystem performance). The performance of the size limit and TAC scenario was almost identical to the TAC scenario and is left out for simplicity.

The management of complex ecosystems is influenced by the tradeoffs of the objectives related to different components of the reef system, i.e., the ecosystem goods (metrics #4) and ecosystem services (metrics #1–3). To show these tradeoffs we present an overall aggregate performance measure kite diagram for the average of the last 5 years of the simulation of each management scenario (Fig 3). Comparing tradeoffs across these scenarios show that each scenario neared the criteria of at least one of the ecosystem metrics (Fig 3). When fishing is regulated according to the integrated ‘full regulation’ scenario (size limit, TAC, MPAs, and no LBSP), landings were reduced to 79% of the status quo landings but all other metrics increased between 115% (biomass herbivores) and 157% (total reef-fish biomass) compared to the status quo (S4 Table). The outcomes of the full regulation scenario approached the criteria for four out of the six metrics (Fig 3). Results were similar for the combined size limit, TAC and no LBSP scenario, but the total reef-fish biomass metric performed less well in this latter scenario and there was one more over-exploited group. This indicates that integrated management including MPAs and off reserve restrictions leads to a higher overall biomass and an increase in the spawning potential compared to open access.

Evaluating the overall performance of each management approach by weighting the management goals 1–4 equally, showed that the scenario with full regulations had the highest average value followed by the combined size limit, TAC and no LBSP scenario (Table 2). Quantifying the performance of just the conservation components (goals 1–3), showed that the full regulation scenario again had the highest overall value (Table 2). With regard to the extraction or socioeconomic components (goal 4), the status quo with no additional LBSP outperformed the other scenarios (0.52), with the size limit scenario being second best (0.48; Table 2).

**Table 2:** Decision table of seven management scenarios based on weightings of performance metrics. Mean normalized results of ecosystem metrics used in performance evaluation of alternative management scenarios with regards to the ecosystem status (goals # 1–3) and socio-economic conditions (metrics # 4a,b). Results for each metric (Table 1, Fig 3) were scaled between zero (worst) and one (best). LBSP is land-based sources of pollution. Bold values are highest (best) values per row.

Decision Table	Status Quo	Size Limit	TAC	TAC & Size Limit	TAC & Size Limit &no LBSP	Status Quo &no LBSP	Full Regula-tions
**Equal weighting of goals (#1–4)**	0.23	0.23	0.45	0.42	0.62	0.46	**0.71**
**Average conservation goals (#1–3)**	0.15	0.14	0.51	0.47	0.68	0.37	**0.75**
**Average extraction goal (#4)**	0.43	0.48	0.47	0.46	0.36	**0.52**	0.46
**Conservation & Extraction equally weighted**	0.29	0.31	0.49	0.46	0.52	0.45	**0.61**

### Alternative fishery regulation scenarios

#### Size-limit scenario

**Fig 4:**
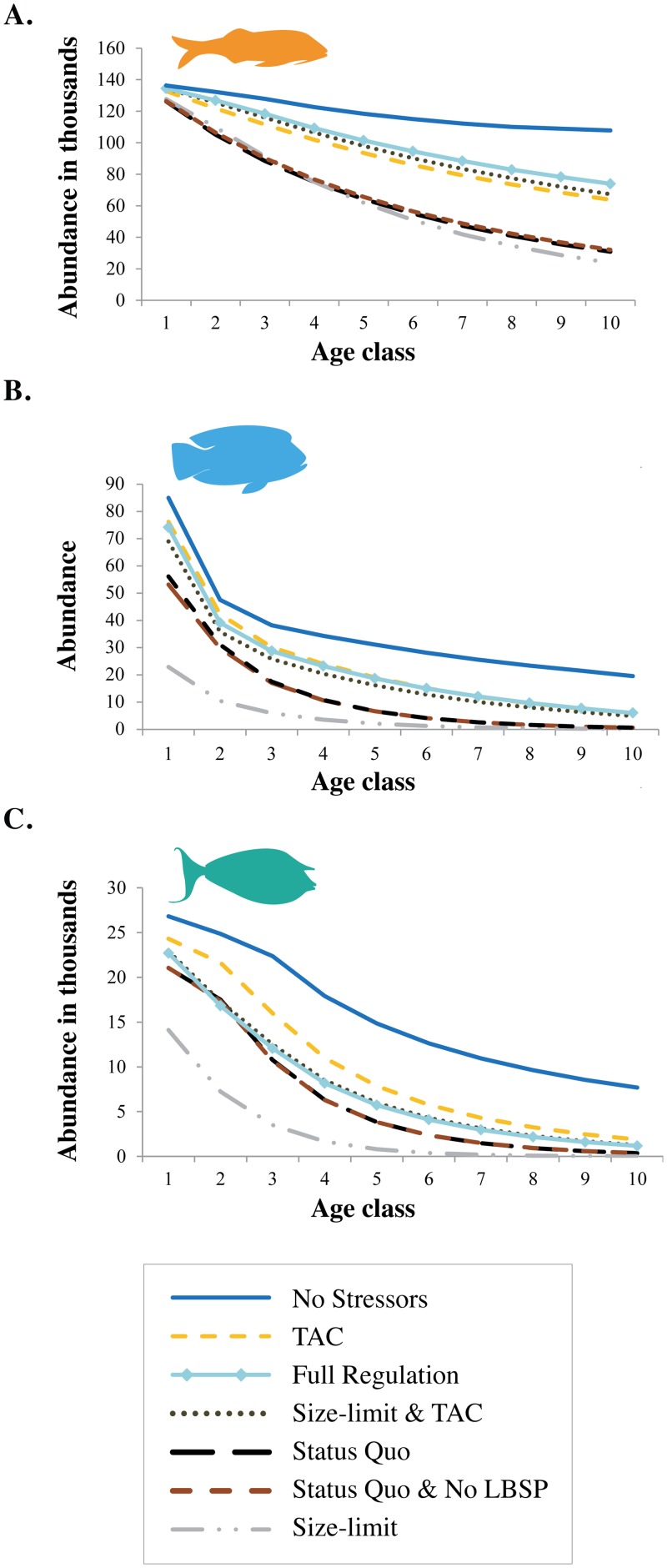
Age-class distributions of (*left*) target invertivorous fish (e.g. goat fish, snapper, wrasse), (*middle*) humphead wrasse and (*right*) target browsers (e.g. unicornfish) at the end of 40 year simulation of different management scenarios. The “No Stressors” scenario indicates no fishing and no land-based sources of pollution (LBSP). The scenario “Size Limit & TAC & no LBSP” had similar results as the “Size Limit & TAC” scenario and is therefore left out for clarity. The “Full Regulation” scenario is comprised of size limit, TAC, MPAs and no LBSP.

For ecosystem state metrics (#1–3) the size-limit based fishery scored slightly less than status quo, but fish landings (goal #4) scored higher (Table 2). The small increase in fish catch, and switch towards targeting larger size classes that is inherent to this scenario, was reflected in the reduction in the abundance of large fishes (Fig 4). None of the ecosystem status metrics reached the criteria (Fig 3, S4 Table) and four fish groups (humphead wrasse (*Cheilinus undulatus*), bumphead parrotfish (*Bolbometopon muricatum*), target browsers (*Naso* sp.) and reef-associated sharks), were overexploited at the end of the simulation, just as in the status quos scenario. Despite the overall reduction in predatory and invertivorous fish functional groups in this scenario, invertebrates themselves did not change noticeably compared to the status quo scenario (Fig 2).

#### TAC scenario

Among fishery regulations scenarios, imposing a TAC led to more favorable outcomes than status quo and size limit scenarios with a tradeoff of 80% of the status quo fish landings. The TAC scenario led to higher biomass of herbivores and overall reef-fish biomass and fewer groups becoming overexploited compared to the status quo and size limit scenarios—only the bumphead parrotfish was still overexploited (Fig 3). Ecosystem effects of the TAC scenario were less pronounced compared to the scenarios with no LBSP, with the main effects being an increase in target invertivores and herbivores (Fig 2).

#### TAC & size limit & no LBSP and full regulation scenarios

The combined size limit, TAC and no LBSP scenario and the full regulation scenario (combined size limit, TAC, no LBSP and MPAs), had respectively two and four metrics that almost reached the criteria (Fig 3), indicating improved ecosystem state compared to the status quo, but at the cost of 80% and 79%, respectively, in status quo fishery landings. The bumphead parrotfish was still overexploited.

### Cumulative effects of climate change

**Fig 5:**
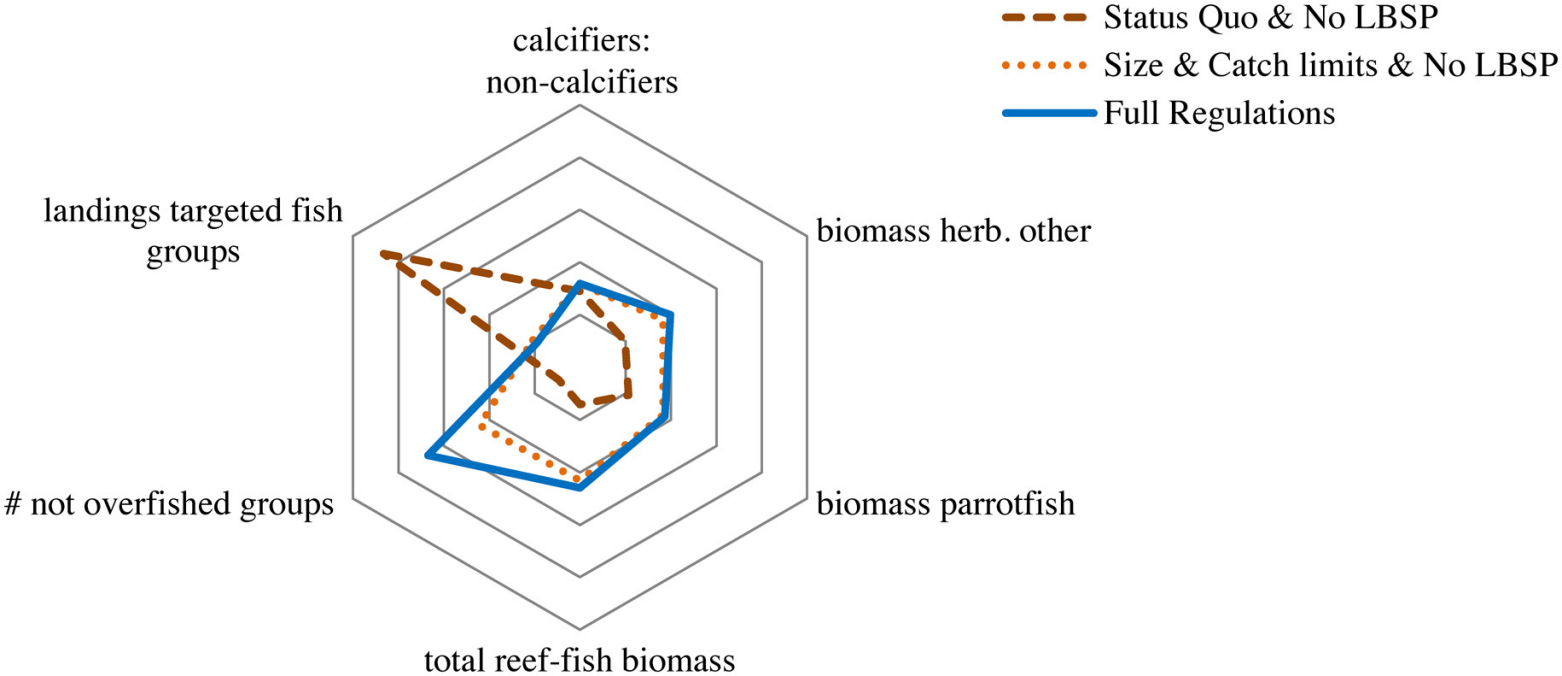
The overall performance of the management strategies including cumulative effects of climate and ocean change for the scaled integrated (composite) performance measures (scaled as in Fig 3). LBSP is land-based sources of pollution (i.e. additional sediments and nutrients).

Cumulative effects of projected climate change (ocean acidification and ocean warming) on corals was simulated for the three management scenarios that performed best overall—all were scenarios with no LBSP: status quo and no LBSP; size limit, TAC and no LBSP; and the full regulation scenarios. Absolute values for the ecosystem metrics varied only slightly between the three scenarios. The most pronounced consequence of incorporating climate change was a large reduction in the ratio of calcifiers to non-calcifiers, to around half of the ratio in scenarios without the simulation of climate change, due to the loss of corals. The overall aggregate performance measure kite diagram shows the tradeoffs of the simulation of each of these three management scenarios with the full regulation and size limit, TAC and no LBSP scenarios being almost identical and scoring higher overall than the status quo and no LBSP on four out of the six performance measures, but again at a costs of the total landings of targeted fish groups (Fig 5).

**Fig 6:**
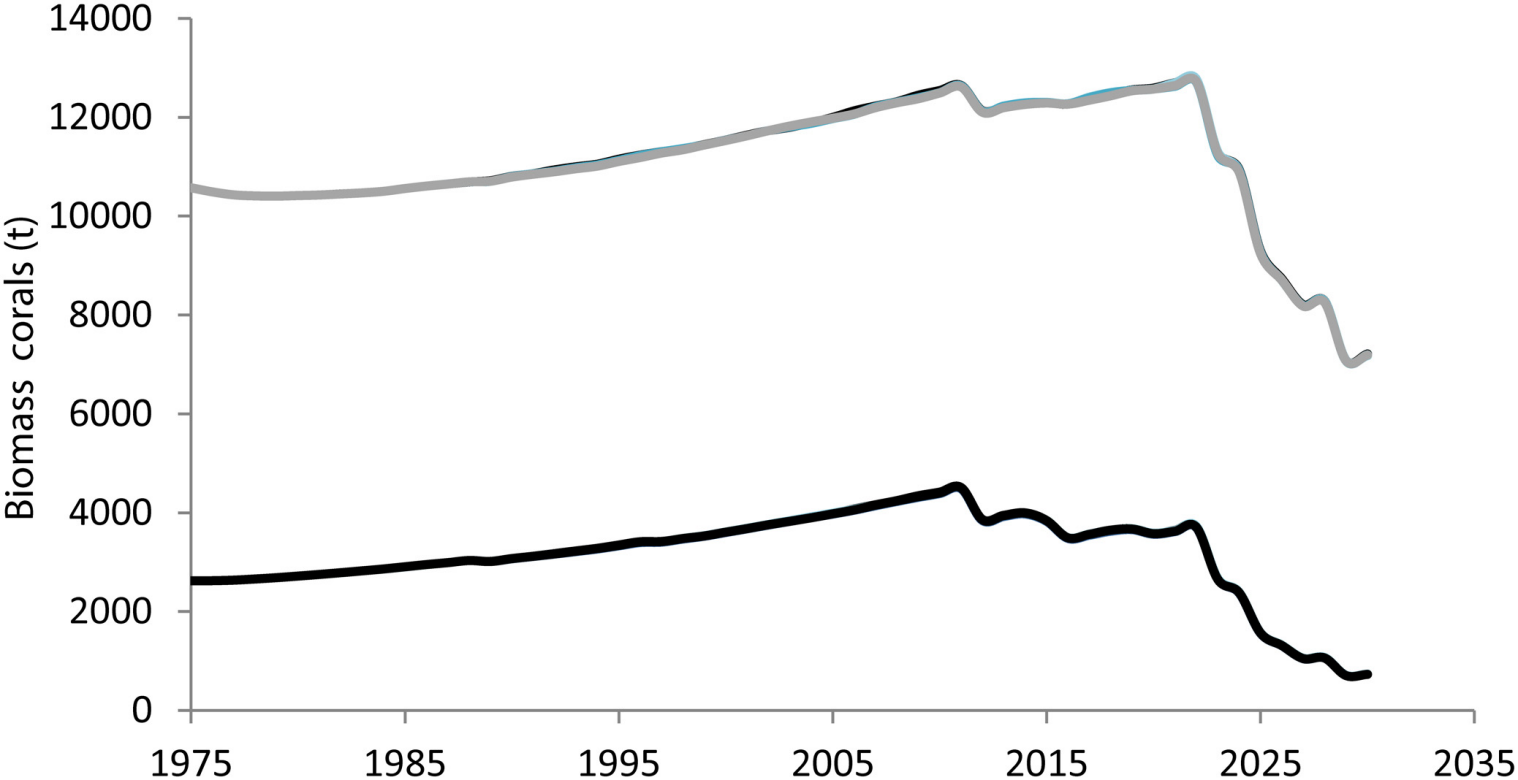
Projected biomass trajectories of (*grey line*) massive corals and (*black line*) branching corals under four fisheries management scenarios, no land-based sources of pollution, and projected climate change impacts (ocean acidification and ocean warming) under the IPCC AR5 RCP8.5 emission scenarios. All simulated scenarios projected the same trend in coral biomass resulting in the overlay of biomass trajectories for branching and massive corals. The scenarios simulated were status quo with no LBSP, size limit and TAC with no LBSP, full regulation, and no fishing and no LBSP (no stressors).

Comparison of outcomes with and without local stressors showed that coral biomass increased in the short-to-medium term (45 years) when stressors are absent. However, the cumulative effects of climate change and local stressors resulted in a sharp reduction in all ecosystem metrics (Fig 5), and coral biomass declined dramatically in 2025, by which time projected ocean temperature regularly (almost annually) surpasses the bleaching threshold and pCO_2_ > 500 ppm (Fig A1 in S1 Text). By that time, corals declined terminally irrespective of which fishery management approach was implemented (Fig 6).

## Discussion

A move toward resilience-based approaches to coral reef management, as an extension of EBM, has been promoted [37]. Empirical evidence and modeling studies have improved understanding of reinforcing feedbacks, hysteresis, and the reversibility of phase-shifts that all influence reef resilience [38–40]. For example, local studies have shown that reversal of an alga-dominated state back to a coral-dominated state is possible on a small scale [41]. Also, when local stressors are reduced [42], for example fishing is reduced [6] or the biomass of herbivores is enhanced [43], corals appear to be more resilient to the effects of bleaching and recover more quickly.

Evaluating selected management strategies in this study showed that each performance metric reached one or more of the criteria while performing less well on other criteria. Only the full regulation scenario, which integrated all management approaches, performed better (closest to outer circle) overall with the main tradeoff being the reduced landings of target reef-fish groups (Fig 3).

MPAs are among the most studied management approaches for coral reef ecosystems [14]. Although MPAs tend to have higher diversity, density and biomass of exploited reef fishes and of some motile invertebrates compared to areas outside of MPAs, and can produce some benefits for reef-associated tourism [44], there is limited evidence that MPAs can be expected to have large impacts outside their boundaries—such as on fisheries yields [44]. Results from our study showed that the status quo scenario (MPAs as the only fishery regulation) did not come out as the ‘best’ overall approach for Guam as a whole. Only for goal 4, maintaining or improving fisheries landings, did status quo produce the ‘best’ result, particularly when there were no land-based sources of pollution. This suggests that to improve ecosystem services, spatial management must be used in conjunction with other forms of fishery regulations (e.g., combined catch limits and size limits), as in the full regulation scenario considered here. In that case, including MPAs does show improved ecosystem services compared with no MPAs (i.e., scenario 6 and 7) in that the overall reef fish biomass is higher and one less functional group was over-exploited. However the ecosystem benefits of using input (size limit) and output (catch limit) controls come at a cost of reduced fishing landings inherent in the reduction in fishing. Such trade-offs need to be acknowledged openly if stakeholder communities are to appreciate the reality of what is needed for reef resilience. Model results were shared with the various stakeholder groups and discussions highlighted the usefulness of the model as a support tool but also in the need to improve the model to address the current model limitations. For example, including the reef-fish landings of the boat-based fishery and scenarios with more realistic reduction of land-based sources of pollution (e.g. 50% reduction instead of the now used 100%) was mentioned.

While the full regulation scenario performed best for the ecosystem state, the cost of enforcement associated with the input and output controls was not factored into our analysis. Enforcing TACs and/or size limits around the entire island is more labor intensive, and hence more costly, than the enforcement of fishery regulations in just site-based marine preserves, as under the current status quo scenario. Moreover enforcement is complicated because fishers typically work on a small scale (e.g., only in areas close to their homes), and hence the area of enforcement is large and therefore costly, and catches include multi species which makes single species management methods expensive. Additionally, success of any fishery regulation is influenced by compliance with these regulations and that depends on the fishers’ costs and revenue associated with illegal fishing but also on moral obligations and the influence of society [45]. Involving stakeholders, including fishers, at an early stage of planning fisheries regulations and education could be a tool to gain acceptance of regulations although education likely will not target all groups and enforcement could still be a necessity to reach objectives [46]. Additional costs related to habitat damage inflicted by fishing gear, abandoned gear (e.g. ghost nets, fishing line), and trampling was also not taken into account [47, 48]. Including the positive habitat effects of marine preserves compared to other fishing gears types in the economic analysis might offset the cost of enforcement [49]. Managers and stakeholders need to agree on the weighting of the contradictory objectives and take into account these issues of gear use and enforcement when making a decision on which management approach would best suit their needs.

When taking into consideration the cumulative effects of climate change impacts, all simulated scenarios performed poorly (Fig 5). Regulations on size-limits and TAC with or without MPAs and no additional LBSP showed a slightly better performance than the status quo scenario with no additional LBSP in terms of fish biomass (total reef fish, herbivores), but that did not correspond with clearly increased capacity for corals to deal with climate change. When the ocean temperature was consistently above the bleaching threshold in successive years and the atmospheric CO_2_ concentration was above 500 ppm, all three approaches showed a severe decline in coral biomass starting as soon as 2025 (Fig 6). This result corresponds with the findings of other recent studies of Pacific coral reef ecosystems [28, 29]. On a global scale, a meta-analysis showed that corals will be in a rapid and terminal decline when the frequency of thermal events is too high for corals to recover [27]. Veron *et al*. [50] showed that corals would be negatively affected by the combined effects of mass bleaching and ocean acidification once CO_2_ concentration reaches above 450 ppm. While Silverman *et al*. [51] suggests that when the CO_2_ concentration surpasses 560 ppm, coral reefs will dissolve, and hence, fisheries management can have little impact on their survival at that point. The results from this case study can be used to draw more general conclusions about the range of management measures that are likely important in practical implementation of EBM in other tropical reefs.

### Model limitations

Ecosystem modeling approaches are becoming more common as a support tool for EBM by providing quantitative evaluations and synthesis of complex dynamics in ecosystems. However, the complexity makes straight forward model skill assessments [52] very hard and in our case the lack of time series made it impossible. Another limitation of the model is that we did not incorporate possible scope for coral adaptation or acclimatization to changing environmental conditions [53], or the effects of cyclones or changes in nutrient supply from deeper waters as a result of predicted increased stratification [54]. Additionally, the physical state of the ecosystem contributed to the uncertainty of model outcomes as the oceanographic module, used to force water flows and the advection of nutrients and plankton, was based on a ROMS model developed for the Coral Triangle (CT-ROMS; Southwest of Guam) rather than targeted on Guam [32]. This meant that Guam was on the ‘edge’ of the CT-ROMS model domain, hence, not adequately incorporating all of the regional oceanographic initial conditions. Lastly, the positive bias in the model towards functional species groups with a high biomass (S1 Text) likely influenced the relative low response of herbivores compared to invertivores (Fig 2) as scrapers, in particular, had a biomass that was 3 times higher than the observed biomass in 2011 [26]. It is likely that the fishing mortality was therefore set too low resulting in a continued increase in the standing stock biomass of scrapers.

Because of the limitations of the model, the results presented here should only be considered relative to each other rather than in absolute terms. The analysis of management options can be considered as a first step and subject to uncertainty that could be resolved (to some degree) in the future by checking the relative performance of the management options across multiple parameterizations of the model.

## Conclusions

Two general conclusions can be drawn from this study. First, choosing among management scenarios with conflicting goals requires a priori weighting of the importance of the various goals. Ecosystem models can be effective tools for local management in visualizing and exploring the costs and benefits of the various approaches under consideration as highlighted in this study. Adoption of the approach that performed best can result in a more effective achievement of socio-ecological goals. Second, under the business as usual greenhouse gas emissions scenario (the RCP8.5 trajectory), with no adaptation or acclimation by reef organisms, the reefs around Guam will collapse in the next few decades. This collapse is likely to occur even with management scenarios in place to alleviate local stressors.

## Supporting Information

S1 Fig Ecosystem response ratio after 45-year simulation of alternative scenarios on (*a*) vertebrates and (*b*) invertebrates (values normalized so 1.0 = best [highest biomass] and 0.0 = worst [lowest biomass]). Results of “Size Limit and TAC” were very similar to only TAC results and left out for clarity. See S2 Table for functional group codes.
(TIF)

S1 Table Participants of informal scoping meetings. (DOCX)

S2 Table Functional groups used in the Guam Atlantis coral reef ecosystem model. (DOCX)

S3 Table Characteristics of reef fisheries per functional group. (DOCX)

S4 Table Results of ecosystem metrics as mean values of last 5 years of a 45-year simulation of seven management scenarios. (DOCX)

S5 Table Data included to create graphs and tables presented in this study. (XLSX)

S1 Text Overview of Guam Atlantis model processes and validation. (DOCX)

S2 Text Justification of selection of performance metrics. (DOCX)
