# Supplementary figures and images for: Management Strategy Evaluation Applied to Coral Reef Ecosystems in Support of Ecosystem-Based Management
Source: PLoS One. 2016 Mar 29;11(3):e0152577. doi: 10.1371/journal.pone.0152577 (PMC4811577; doi:10.1371/journal.pone.0152577)

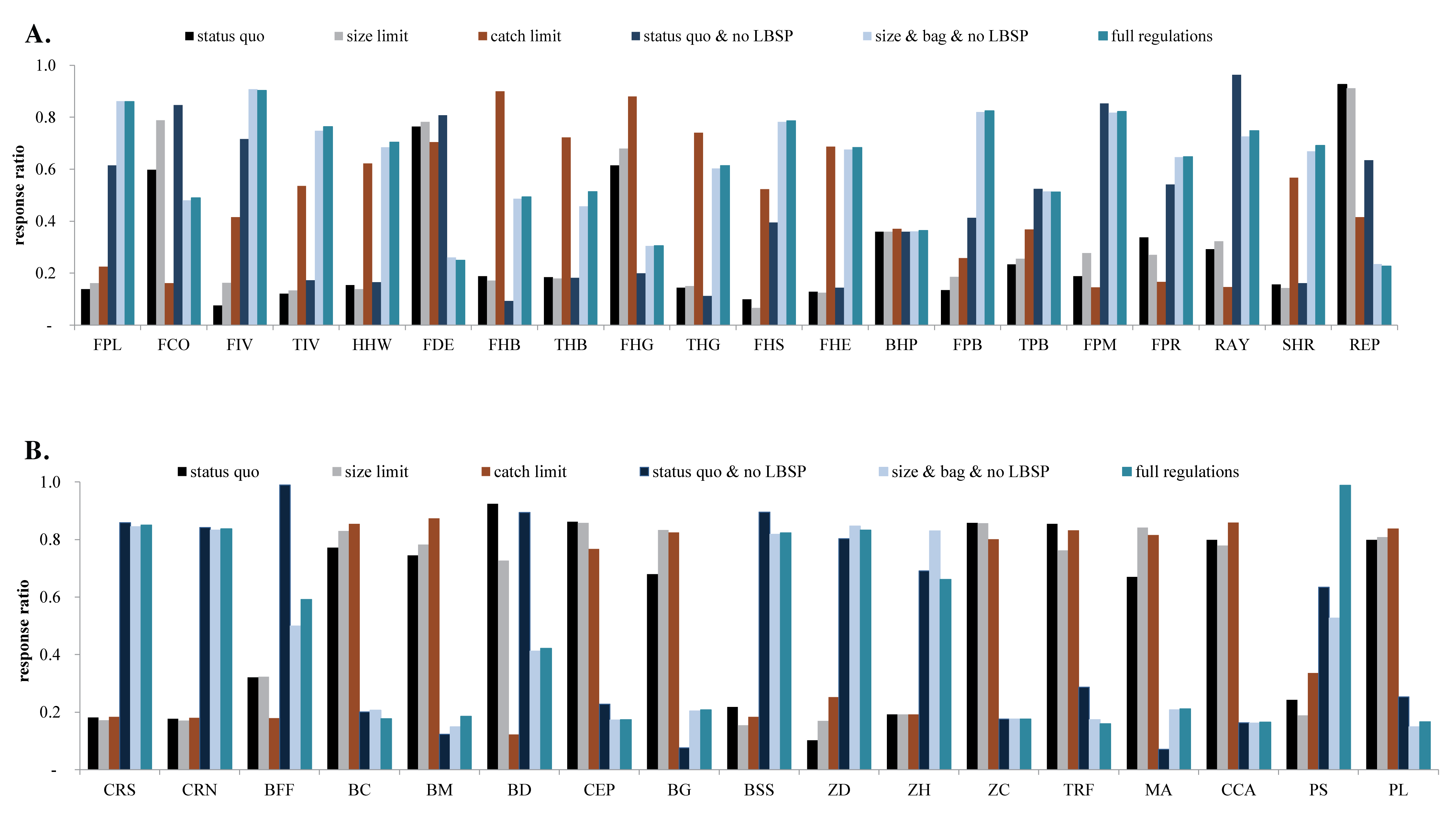

Supplement: S1 Fig — Results of “Size Limit and TAC” were very similar to only TAC results and left out for clarity. See S2 Table for functional group codes. (TIF) [file pone.0152577.s001.tif]
