# Supplementary material for: Management Strategy Evaluation Applied to Coral Reef Ecosystems in Support of Ecosystem-Based Management
Source: PLoS One. 2016 Mar 29;11(3):e0152577. doi: 10.1371/journal.pone.0152577 (PMC4811577; doi:10.1371/journal.pone.0152577)
Supplement: S1 Table — (DOCX) [file pone.0152577.s002.docx]

# S1 Table. Organizations represented at informal meetings to discuss ecosystem modeling as a decision-support tool.

Based on the regulations of the University of Hawaii Office of Research Compliance no Institutional Review Board application was needed since human involvement was not part of a structured, systematic investigation nor did we obtain any health or personal information from the informal meeting participants. Stakeholders received an invitation to attend the informal meeting. Participation was on a voluntary basis.

| Participants Workshop Nov. 2012 | No. of people | Participants Follow-up meetings Nov 2012 | No. of people |
| --- | --- | --- | --- |
| NOAA Coral Reef Conservation Program (CRCP) | 2 | Guam POC CRCP, West Pacific Management Fishery Council, President of Dept. of Chamorro Affairs | 1 |
| NOAA Pacific Island Regional Office | 1 | Guam Environmental Protection Agency | 2 |
| Guam Coastal Management Program | 2 | Guam Coastal Management Program (GCMP) | 3 |
| NOAA Pacific Islands Fisheries Science Center | 1 | War in the Pacific National Park (NPS) | 3 |
| Naval Facility Andersen Air Force Base (AAFB) | 3 | University of Guam, Marine Lab | 4 |
| USDA Natural Resource Conservation Service | 1 |  |  |
| University of Guam, Marine Lab | 1 |  |  |
| Humatak Community Foundation | 1 |  |  |
| Guam Preservation Trust | 1 |  |  |
| Dept. of Agriculture | 1 |  |  |
| Guam POC CRCP, West Pacific Management Fishery Council, President of Dept. of Chamorro Affairs | 1 |  |  |
