# Supplementary material for: Management Strategy Evaluation Applied to Coral Reef Ecosystems in Support of Ecosystem-Based Management
Source: PLoS One. 2016 Mar 29;11(3):e0152577. doi: 10.1371/journal.pone.0152577 (PMC4811577; doi:10.1371/journal.pone.0152577)
Supplement: S2 Table — (DOCX) [file pone.0152577.s003.docx]

# S2 Table. Functional groups used in the Guam Atlantis coral reef ecosystem model.

Groups with an * are exploited in the recreational shore-based fishery.

| **#** | **Code** | **Group** | **Common name** | **Family** | **Scientific name** | **Biomass (t/km^2^)** | | **% of total group** | | **cummula-tive sum** | |
| --- | --- | --- | --- | --- | --- | --- | --- | --- | --- | --- | --- |
|  | **FISH** |  |  |  |  | *(wet weight) Italics is tow data; Bold is total per group* | |  |  |  |  |
| **1** | **FPL** | **Planktivores** | | |  | **2.31** |  | |  | |  |
|  |  |  | Ocellate damselfish | Pomacentridae | *Pomacentrus vaiuli* | 0.33 | 14.4 | | 0.14 | |  |
|  |  |  | Shoulderbar soldierfish | Holocentridae | *Myripristis kuntee* | 0.32 | 13.9 | | 0.28 | |  |
|  |  |  | Whitespotted devil | Pomacentridae | *Plectroglyphidodon lacrymatus* | 0.18 | 7.8 | | 0.36 | |  |
|  |  |  | Redtoothed triggerfish | Balistidae | *Odonus niger* | 0.10 | 6.9 | | 0.43 | |  |
|  |  |  | Guam damsel | Pomacentridae | *Pomachromis guamensis* | 0.16 | 6.8 | | 0.50 | |  |
|  |  |  | Midnight snapper | Lutjanidae | *Macolor macularis* | 0.15 | 6.4 | | 0.56 | |  |
|  |  |  | Black and white snapper | Lutjanidae | *Macolor niger* | 0.09 | 3.8 | | 0.60 | |  |
|  |  |  | Blenny species | Blenniidae | *Blenniidae* | 0.07 | 2.9 | | 0.63 | |  |
|  |  |  | Blotcheye soldierfish | Holocentridae | *Myripristis berndti* | 0.06 | 2.6 | | 0.66 | |  |
|  |  |  | Chinese zebra goby | Microdesmidae | *Ptereleotris zebra* | 0.05 | 2.1 | | 0.68 | |  |
|  |  |  | Sleek unicornfish | Acanthuridae | *Naso hexacanthus* | 0.05 | 2.0 | | 0.70 | |  |
|  |  |  | Reticulate dascyllus | Pomacentridae | *Dascyllus reticulatus* | 0.05 | 2.0 | | 0.72 | |  |
|  |  |  | Midget chromis | Pomacentridae | *Chromis acares* | 0.04 | 1.9 | | 0.73 | |  |
|  |  |  | Red shoulder wrasse | Labridae | *Stethojulis bandanensis* | 0.04 | 1.8 | | 0.75 | |  |
| **2** | **FCO** | **Coralivores** | |  |  | **0.29** |  | |  | |  |
|  |  |  | Mailed butterflyfish | Chaetodontidae | *Chaetodon reticulatus* | 0.07 | 25.8 | | 0.26 | |  |
|  |  |  | Speckled butterflyfish | Chaetodontidae | *Chaetodon citrinellus* | 0.06 | 19.1 | | 0.45 | |  |
|  |  |  | Threeband pennantfish | Chaetodontidae | *Heniochus chrysostomus* | 0.03 | 10.0 | | 0.55 | |  |
|  |  |  | Johnston Island damsel | Pomacentridae | *Plectroglyphidodon johnstonianus* | 0.03 | 10.3 | | 0.66 | |  |
|  |  |  | Oval butterflyfish | Chaetodontidae | *Chaetodon lunulatus* | 0.03 | 9.7 | | 0.75 | |  |
| **3** | **FIV** | **Invertivores** | | | | **1.748** |  | |  | |  |
|  |  |  | Orange-lined triggerfish | Balistidae | *Balistapus undulatus* | 0.23 | 13.3 | | 0.13 | |  |
|  |  |  | Blackbar devil | Pomacentridae | *Plectroglyphidodon dickii* | 0.23 | 12.9 | | 0.26 | |  |
|  |  |  | Arc-eye hawkfish | Cirrhitidae | *Paracirrhites arcatus* | 0.19 | 10.6 | | 0.37 | |  |
|  |  |  | Halfmoon triggerfish | Balistidae | *Sufflamen chrysopterum* | 0.14 | 7.9 | | 0.44 | |  |
|  |  |  | Boomerang triggerfish | Balistidae | *Sufflamen bursa* | 0.13 | 6.7 | | 0.51 | |  |
|  |  |  | Blueband goby | Gobiidae | *Valenciennea strigata* | 0.07 | 4.3 | | 0.55 | |  |
|  |  |  | Red-lined wrasse | Labridae | *Halichoeres biocellatus* | 0.07 | 4.2 | | 0.60 | |  |
|  |  |  | Moorish idol | Zanclidae | *Zanclus cornutus* | 0.06 | 3.8 | | 0.63 | |  |
|  |  |  | Royal angelfish | Pomacanthidae | *Pygoplites diacanthus* | 0.06 | 3.6 | | 0.67 | |  |
|  |  |  | Raccoon butterflyfish | Chaetodontidae | *Chaetodon lunula* | 0.06 | 3.4 | | 0.70 | |  |
|  |  |  | Latticed sandperch | Pinguipedidae | *Parapercis clathrata* | 0.04 | 2.1 | | 0.73 | |  |
|  |  |  | Titan triggerfish | Balistidae | *Balistoides viridescens* | 0.04 | 2.1 | | 0.75 | |  |
| **4** | **TIV** | **Target Invertivores*** | | | | **2.213** |  | |  | |  |
|  |  |  | Humpnose big-eye bream | Lethrinidae | *Monotaxis grandoculis* | 0.50 | 22.7 | | 0.23 | |  |
|  |  |  | Fivestripe wrasse | Labridae | *Thalassoma quinquevittatum* | 0.41 | 18.4 | | 0.41 | |  |
|  |  |  | Manybar goatfish | Mullidae | *Parupeneus multifasciatus* | 0.16 | 7.3 | | 0.48 | |  |
|  |  |  | Striped large-eye bream | Lethrinidae | *Gnathodentex aureolineatus* | 0.10 | 4.6 | | 0.53 | |  |
|  |  |  | Silverspot squirrelfish | Holocentridae | *Sargocentron caudimaculatum* | 0.08 | 3.8 | | 0.57 | |  |
|  |  |  | Common bluestripe snapper | Lutjanidae | *Lutjanus kasmira* | 0.06 | 3.0 | | 0.60 | |  |
|  |  |  | Checkerboard wrasse | Labridae | *Halichoeres hortulanus* | 0.07 | 3.0 | | 0.63 | |  |
|  |  |  | wrasse | Labridae | *Cheilinus sp* | 0.06 | 2.5 | | 0.65 | |  |
|  |  |  | Blacktail snapper | Lutjanidae | *Lutjanus fulvus* | 0.06 | 2.5 | | 0.68 | |  |
|  |  |  | Blue lined squirrelfish | Holocentridae | *Sargocentron tiere* | 0.05 | 2.4 | | 0.70 | |  |
|  |  |  | Tripletail wrasse | Labridae | *Cheilinus trilobatus* | 0.05 | 2.2 | | 0.72 | |  |
|  |  |  | Pastel ringwrasse | Labridae | *Hologymnosus doliatus* | 0.05 | 2.1 | | 0.74 | |  |
|  |  |  | Sammara squirrelfish | Holocentridae | *Neoniphon sammara* | 0.05 | 2.1 | | 0.77 | |  |
| **5** | **HHW** | **Humphead wrasse*** | |  |  | **0.017** |  | |  | |  |
|  |  |  | Humphead/Napolean wrasse | Labridae | *Cheilinus undulatus* | 0.02 | 1 | | 1 | |  |
| **6** | **FDE** | **Detritivores** | | | | **1.42** |  | |  | |  |
|  |  |  | Striated surgeonfish | Acanthuridae | *Ctenochaetus striatus* | 1.34 | 93.9 | | 0.94 | |  |
| **7** | **FHB** | **Browsers** | |  |  | **0.06** |  | |  | |  |
|  |  |  | Carolines parrotfish | Scaridae | *Calotomus carolinus* | 0.05 | 94.8 | | 0.95 | |  |
| **8** | **THB** | **Target Browsers*** | | |  | **0.89** |  | |  | |  |
|  |  |  | Orangespine unicornfish | Acanthuridae | *Naso lituratus* | 0.58 | 65.6 | | 0.66 | |  |
|  |  |  | Bulbnose unicornfish | Acanthuridae | *Naso tonganus* | 0.07 | 7.9 | | 0.73 | |  |
|  |  |  | Chubs | Kyphosidae | *Kyphosus sp* | 0.05 | 5.6 | | 0.79 | |  |
| **9** | **FHG** | **Grazers** | | |  | **1.26** |  | |  | |  |
|  |  |  | Pinktail triggerfish | Balistidae | *Melichthys vidua* | 0.51 | 40.4 | | 0.40 | |  |
|  |  |  | Pacific gregory | Pomacentridae | *Stegastes fasciolatus* | 0.30 | 23.5 | | 0.64 | |  |
|  |  |  | Surge damselfish | Pomacentridae | *Chrysiptera brownriggii* | 0.22 | 17.1 | | 0.81 | |  |
| **10** | **THG** | **Target Grazers*** | |  |  | **2.33** |  | |  | |  |
|  |  |  | Brown surgeonfish | Acanthuridae | *Acanthurus nigrofuscus* | 0.96 | 41.0 | | 0.41 | |  |
|  |  |  | Lined surgeonfish | Acanthuridae | *Acanthurus lineatus* | 0.74 | 31.6 | | 0.73 | |  |
|  |  |  | Whitecheek surgeonfish | Acanthuridae | *Acanthurus nigricans* | 0.23 | 9.7 | | 0.82 | |  |
|  |  |  | |  |  |  |  | |  | |  |
| **11** | **FHS** | **Scrapers*** | |  |  | **3.66** |  | |  | |  |
|  |  |  | Daisy parrotfish | Scaridae | *Chlorurus sordidus* | 2.29 | 62.5 | | 0.62 | |  |
|  |  |  | Common parrotfish | Scaridae | *Scarus psittacus* | 0.61 | 16.6 | | 0.79 | |  |
| **12** | **FHE** | **Excavators*** | |  |  | **0.62** |  | |  | |  |
|  |  |  | Tan-faced parrotfish | Scaridae | *Chlorurus frontalis* | 0.26 | 42.4 | | 0.42 | |  |
|  |  |  | Filament-finned parrotfish | Scaridae | *Scarus altipinnis* | 0.24 | 38.7 | | 0.81 | |  |
|  |  |  | Ember parrotfish | Scaridae | *Scarus rubroviolaceus* | 0.12 | 18.9 | | 1 | |  |
| **13** | **BHP** | **Bumphead parrotfish*** (none seen during visual surveys, biomass assumed) | | | | **0.01** |  | |  | |  |
|  |  |  | Humphead parrotfish | Scaridae | *Bolbometopon muricatum* | 0.01 | 1 | | 1 | |  |
| **14** | **FPB** | **Benthic piscivores** | | |  | **0.14** |  | |  | |  |
|  |  |  | Blackside hawkfish | Cirrhitidae | *Paracirrhites forsteri* | 0.06 | 44.4 | | 0.44 | |  |
|  |  |  | Whitespot hawkfish | Cirrhitidae | *Paracirrhites hemistictus* | 0.03 | 25.1 | | 0.70 | |  |
|  |  |  | Giant moray | Muraenidae | *Gymnothorax javanicus* | 0.02 | 11.8 | | 0.81 | |  |
| **15** | **TPB** | **Target benthic piscivores*** | | | | **1.09** |  | |  | |  |
|  |  |  | Darkfin hind | Serranidae | *Cephalopholis urodeta* | 0.30 | 27.4 | | 0.27 | |  |
|  |  |  | Ringtail maori wrasse | Labridae | *Oxycheilinus unifasciatus* | 0.23 | 21.1 | | 0.49 | |  |
|  |  |  | Peacock hind | Serranidae | *Cephalopholis argus* | 0.16 | 14.4 | | 0.63 | |  |
|  |  |  | Blacktip grouper | Serranidae | *Epinephelus fasciatus* | 0.11 | 10.3 | | 0.73 | |  |
|  |  |  | Two-spot red snapper | Lutjanidae | *Lutjanus bohar* | 0.10 | 9.2 | | 0.82 | |  |
| **16** | **FPM** | **Mid-water piscivores*** | | | | **0.46** |  | |  | |  |
|  |  |  | Small toothed jobfish | Lutjanidae | *Aphareus furca* | 0.45 | 99.3 | | 0.99 | |  |
| **17** | **FPR** | **Roving piscivores*** | |  |  | ***1.09*** |  | |  | |  |
|  |  |  | Blackfin barracuda | Sphyraenidae | *Sphyraena qenie* | *0.60* | 55.1 | | 0.55 | |  |
|  |  |  | Jacks | Carangidae | *Caranx sp* | *0.40* | 36.7 | | 0.92 | |  |
|  | **SHARKS** |  |  |  |  |  |  | |  | |  |
| **18** | **SHR** | **Reef-associated sharks*** | |  |  | ***0.15*** |  | |  | |  |
|  |  |  | Tawny nurse shark | Carcharhinidae | *Nebrius ferrugineus* | *0.09* | 58.0 | | 0.58 | |  |
|  |  |  | Whitetip reef shark | Carcharhinidae | *Triaenodon obesus* | *0.04* | 26.0 | | 0.84 | |  |
| **19** | **RAY** | **Rays*** |  |  |  | ***0.13*** |  | |  | |  |
|  |  |  | Spotted Eagle ray | Myliobatidae | *Aetobatus narinari* | *0.08* | 66.3 | | 0.66 | |  |
|  |  |  | Porcupine ray | Dasyatidae | *Urogymnus asperrimus* | *0.04* | 33.7 | | 1 | |  |
|  | **REPTILES** | |  |  |  |  |  | |  | |  |
| **20** | **REP** | **Sea Turtles** | | | | **0.80** |  | |  | |  |
|  |  |  | Green turtle | Chelonidae | *Chelonia mydas* | 0.80 | 1 | | 1 | |  |

| **Code** | **Group** | **species** | | | | **Biomass** | | **Source biomass data** | | |
| --- | --- | --- | --- | --- | --- | --- | --- | --- | --- | --- |
| **INVERTEBRATES** |  |  | | | |  | | |  |  |
| BC | Benthic Carnivores* | carnivorous gastropods, crusteaceans and polycheates | | | | 20.13 | | | CRED, UoG, CMP |  |
| BD | Benthic Detritivores* | sea cucumbers, lobster, polycheates, detritivorous gastropods and crusteaceans (e.g. crabs) | | | | 17.10 | | | CRED, UoG, CMP |  |
| BM | Benthic Meiofauna | infauna: small herbivorous polychaetes, gastropods and crustaceans | | | | 10.97 | | | CRED, UoG, CMP |  |
| BFF | Benthic Suspension Feeders* | octocoral, sponges, tunicates, zooanthids, giant clams, bivalves, polychaetes, foraminifera, bryzoans, brittle stars | | | | 216.53 | | | CRED, UoG, CMP |  |
| CRS | Sheltering corals | branching/tabular morphology | | | | 30.73 | | | CRED, UoG, CMP |  |
| CRN | Non-sheltering corals | massive/encrusting morphology | | | | 108.92 | | | CRED, UoG, CMP |  |
| CEP | Cephalopods* | octopus, squids | | | | 1.00 | | | CRED, UoG, CMP |  |
| BG | Benthic Grazers* | urchins (helmet, collectors, pencil, boring, diadema) | | | | 0.16 | | | CRED, UoG, CMP |  |
| BSS | Sea Stars | including crown-of-thorns seastar | | | | 0.21 | | | CRED, UoG, CMP |  |
| **ALGAE** |  |  |  |  |  |  |  |  |  |  |
| TRF | Turf algae | < 1cm | | | | 199.13 | | | CRED |  |
| MA | Macroalgae* | > 1cm | | | | 188.65 | | | CRED |  |
| CCA | Crustose-coraline algae |  | | | | 102.53 | | | CRED |  |
| **PLANKTON** | |  | | | |  |  | |  |  |
| PS | Small phytoplankton | picoeukaryotes, cyanobacteria, < 1um | | | | 0.16 | | | CRED, ([Wang et al., 2008](#_ENREF_6)) |  |
| PL | Large phytoplankton | incl. diatoms | | | |  | | | CRED, ([Wang et al., 2008](#_ENREF_6)) |  |
| ZC | Zooplankton -carnivores | chaetognath, amphipods, crab larvae, isopods, mysid shrimps, polychaetes (micronekton) | | | | 0.8 | | | ([Hamner et al., 2007](#_ENREF_4); [Suntsov and Domokos, 2013](#_ENREF_5)) |  |
| ZD | Demersal zooplankton | pelagic fish & invert larvae, copepods, polychaetes, foraminiferas | | | | 1.5 | | | ([Alldredge and King, 2009](#_ENREF_2)), 15 times oceanic zooplankton ([Alldredge and King, 1977](#_ENREF_1)) density varies with live coral ([Grimm and Clayshulte, 1981](#_ENREF_3)) |  |
| ZH | Zooplankton - herbivores | copepods, mesonekton | | | | 0.36 | | | ([Suntsov and Domokos, 2013](#_ENREF_5))([Hamner et al., 2007](#_ENREF_4)) |  |
| **BACTERIA** | |  | | | |  |  | |  |  |
| PB | Pelagic Bacteria | hetrotrophic bacteria (0.2-1 um) | | | |  | | | CRED |  |
| BB | Benthic bacteria | hetrotrophic bacteria (0.2-1 um) | | | |  | | |  |  |
| **DETRITUS** | |  | | | |  |  | |  |  |
| DC | carrion | dead | | | | 0 | | |  |  |
| DR | refractory detritus | long 'life' time | | | |  | | | EPA |  |
| DL | Labile detritus | easily degraded | | | |  | | | EPA |  |

**References for Appendix 3**

Alldredge, A., and King, J. 1977. Distribution, abundance, and substrate preferences of demersal reef zooplankton at Lizard Island Lagoon, Great Barrier Reef. Marine Biology, 41: 317-333.

Alldredge, A., and King, J. 2009. Near-surface enrichment of zooplankton over a shallow back reef: implications for coral reef food webs. Coral Reefs, 28: 895-908.

Grimm, G. R., and Clayshulte, R. N. 1981. Demersal plankton from Western Shoals, Apra Harbor, Guam. *In* Proceedings of 4th International Coral Reef Symposium, p. 454. Ed. by E. Gomez, C. Birkeland, R. Buddemeier, R. Johannes, J. Marsh Jr., and R. Tsuda. Marine Sciences Center, University of the Philippines, Manila, Philippines.

Hamner, W. M., Colin, P. L., and Hamner, P. P. 2007. Export-import dynamics of zooplankton on a coral reef in Palau. Marine Ecology Progress Series, 334: 83-92.

Suntsov, A., and Domokos, R. 2013. Vertically migrating micronekton and macrozooplankton communities around Guam and the Northern Mariana Islands. Deep Sea Research Part I: Oceanographic Research Papers, 71: 113-129.

Wang, X., Behrenfeld, M., Le Borgne, R., Murtugudde, R., and Boss, E. 2008. Regulation of phytoplankton carbon to chlorophyll ratio by light, nutrients and temperature in the Equatorial Pacific Ocean: a basin-scale model. Biogeosciences, 5: 3869-3903.
