# Supplementary material for: Management Strategy Evaluation Applied to Coral Reef Ecosystems in Support of Ecosystem-Based Management
Source: PLoS One. 2016 Mar 29;11(3):e0152577. doi: 10.1371/journal.pone.0152577 (PMC4811577; doi:10.1371/journal.pone.0152577)
Supplement: S3 Table — (DOCX) [file pone.0152577.s004.docx]

S3 Table. Characteristics of reef fisheries per functional fish group. Fishing was modeled using constant instantaneous fishing mortality rates using mortality rates as shown in the first column, based on historical catches and estimates of biomass. For the size-limited fishery scenario the size and age at first capture for each functional group were set equal to the size and age at first maturity from published sources.

| **Functional group** | **Fishing mortality (% per year)** | **Age at first maturity (years)** | **Length at first maturity (cm)** | **Weight at first maturity (g)** | **Age when fishery starts in model** | **Source life-history information (FishBase with additional data indicated by citation)** | |
| --- | --- | --- | --- | --- | --- | --- | --- |
| Planktivores | 0.026 | 1.2 | 13.9 | 162 | 2 | {Dee, 1994 #93;Wilson, 1999 #696} |  |
| Coralivores | 0.0001 | 1.2 | 10.7 | 177 | 2 | {MacDonald, 1981 #776} |  |
| Invertivores | 0.033 | 1.4 | 15.9 | 199 | 2 | FishBase |  |
| Target invertivores | 0.200 | 2.2 | 22.0 | 258 | 2 | {Taylor, 2012 #765} |  |
| Humphead wrasse | 0.218 | 6.0 | 70 | 6,827 | 6 | {Choat, 2006 #768} |  |
| Detritivores | 0.001 | 0.9 | 13.3 | 64 | 2 | FishBase |  |
| Browsers | 0.116 | 1.1 | 20.2 | 670 | 4 | {Taylor, 2012 #765} |  |
| Target browsers | 1.075 | 2.6 | 20.6 | 352 | 2 | {Taylor, 2012 #765} |  |
| Grazers | 0.004 | 1.7 | 14.0 | 193 | 3 | FishBase |  |
| Target grazers | 0.068 | 1.4 | 13.7 | 79 | 2 | {Hart, 1996 #769} |  |
| Scrapers | 0.018 | 1.4 | 17.9 | 84 | 2 | {Taylor, 2012 #765} |  |
| Excavators | 0.157 | 2.2 | 25.3 | 342 | 2 | {Taylor, 2012 #765} |  |
| Bumphead parrotfish | 1.50 | 8.0 | 61.0 | 4696 | 6 | {Hamilton, 2008 #770} |  |
| Benthic piscivores | 0.096 | 2.0 | 76.5 | 996 | 2 | FishBase |  |
| Target benthic piscivores | 0.041 | 3.8 | 25.8 | 538 | 4 | {Rhodes, 2011 #772} |  |
| Mid-water piscivores | 0.187 | 2.0 | 39.3 | 626 | 2 | FishBase |  |
| Roving piscivores | 0.104 | 5.6 | 77.1 | 2708 | 6 | {Sudekum, 1991 #289;Longenecker, 2008 #774} |  |
| Reef sharks | 0.266 | 4.3 | 112.0 | 21,591 | 4 | FishBase |  |
| Rays | 0.003 | 5.4 | 150 | 38,196 | 4 | {Schluessel, 2008 #784} |  |
| Turtles* | <0.0001 | 37.5 | 81 | 82,414 | 36 | {Frazer, 1985} and Ehrhart 1985, {Balazs, 2004} |  |

* turtles are not reported in catch data (take is prohibited) but anecdotal evidence suggests that they are served at large cultural events so we assumed a low harvest rate
