# Supplementary material for: Management Strategy Evaluation Applied to Coral Reef Ecosystems in Support of Ecosystem-Based Management
Source: PLoS One. 2016 Mar 29;11(3):e0152577. doi: 10.1371/journal.pone.0152577 (PMC4811577; doi:10.1371/journal.pone.0152577)
Supplement: S4 Table — (DOCX) [file pone.0152577.s005.docx]

S4 Table. Results of ecosystem metrics as mean values of last 5 years of a 45-year simulation of seven management scenarios. The criteria are the mean values of a 45-year simulation of no fishing and no land-based sources of pollution (LBSP). Calc is calcifiers (corals and crustose-coralline algae); non-calc is non-calcifiers (turf and macro-algae). Target fish groups are fish groups targeted by shore-based fishers (Table A1, Appendix 5).

|  | **Status Quo** | **Size-limit** | **TAC** | **Size**  **limit &**  **TAC** | **Status Quo & No LBSP** | **Size limit & TAC &No LBSP** | **Full regula-tions** | ***Criteria*** |
| --- | --- | --- | --- | --- | --- | --- | --- | --- |
| ratio calc.: non-calc. | 0.79 | 0.76 | 0.77 | 0.76 | 0.98 | 0.94 | 0.94 | *0.94* |
| biomass herb. other | 988 | 990 | 1,220 | 1,189 | 1,122 | 963 | 1,134 | *1,344* |
| biomass parrotfish | 844 | 838 | 958 | 952 | 972 | 870 | 974 | *1,043* |
| total reef-fish biomass | 3,412 | 3,445 | 4,154 | 4,114 | 4,654 | 3,823 | 5,356 | *5,309* |
| # not overfished groups | 16 | 16 | 19 | 19 | 18 | 16 | 19 | *20* |
| landings targeted fish groups | 128 | 133 | 102 | 100 | 103 | 141 | 101 | *128* |
