# Supplementary material for: Management Strategy Evaluation Applied to Coral Reef Ecosystems in Support of Ecosystem-Based Management
Source: PLoS One. 2016 Mar 29;11(3):e0152577. doi: 10.1371/journal.pone.0152577 (PMC4811577; doi:10.1371/journal.pone.0152577)
Supplement: S1 Text — (DOCX) [file pone.0152577.s007.docx]

# S1 Text. Overview of Guam Atlantis model processes and validation.

Guam Atlantis is based on the Atlantis framework developed at CSIRO Australia [[1-3](#_ENREF_1)] and includes a recently developed coral module [[4](#_ENREF_4)], which was parameterized for the coral reef ecosystem around Guam. The coral module is based on key coral reef dynamics identified from a literature review and the form of the relationships for those dynamics [[4](#_ENREF_4)]. Detailed in the coral module are the dynamics of coral growth (and growth-related complexity) and competition with benthic algae that are influenced by three main drivers: (1) land-based sources of pollution (LBSP, a local stressor); (2) fishing activities (also a local stressor); and climate change (a global stressor). By including extensive empirical data collected from field studies in Guam, local-scale dynamics are projected over decades, and trends that will likely manifest themselves locally are identified.

Land-based sources of pollution (LBSP) were modeled as additional input of nitrogen and sediments into coastal polygons that had riverine runoff or sewage outflow pipes [[5](#_ENREF_5)]. The sediment and nutrient loads were based on data collected from 2005–2011 (Guam Environmental Protection Agency, War-of-the-Pacific National Park and NOAA Pacific Islands Fisheries Science Center, Coral Reef Ecosystem Program) and used as initial condition input data for the model. River flow and additional nutrient and sediment input data were based on outflow time series from 1991 and 2011 (USGS data) and the last year was repeated for future projections. These outflow time series did not show any temporal trend and, for simplicity, we assumed no future changes in land-use or the amount of rain fall (and hence river out flow).

Fishing was modeled as a fixed fishing mortality in each spatial polygon around Guam except for marine protected areas (MPAs). To set our fixed fishing mortality, we first calibrated the model to spatial explicit historical landings of shore-based fishery and biomass trends [[5](#_ENREF_5), [6](#_ENREF_6)] and calculated the fishing mortality for each functional species group by dividing the landings at year 1 with the standing stock biomass at year 0 to get a proxy for annual fishing mortality for that functional group. We then used those proxy values as the fixed fishing mortality per functional group.

For climate change we modeled ocean acidification and ocean warming. Ocean acidification negatively affected growth rates of corals as well as echinoderms, molluscs, crustose coralline algae and copepods (modeled as herbivorous zooplankton), and increased the growth rates of phytoplankton and macroalgae [[7-10](#_ENREF_7)]. Time series of atmospheric CO_2_ came from the International Panel on Climate Change Assessment Report 5 using the highest emission scenario Representative Concentration Pathway (RCP) 8.5 projection. These time series data were read into Atlantis to calculate the change in pH and aragonite saturation state and, ultimately, therefore calcification rates of corals and other calcifiers [[11-13](#_ENREF_11)]. Effects of ocean warming included coral bleaching (the expulsion of algal symbionts from the coral tissue) leading to lower coral growth rates and higher mortality. Bleaching events were triggered when ocean temperature exceeded the local bleaching threshold (1°C above the maximum summer temperature in Guam) for more than specified periods of time (known as ‘degree heating weeks’ [DHW] that includes the magnitude and duration of elevated temperatures) [[14-16](#_ENREF_14)]. Predicted sea surface temperature data came from the RCP8.5 projection using the HadGEM-AO model output, which has been shown to perform well compared to 20 similar models from the Coupled Model Intercomparison Project Phase5 [CMIP5] (CRED unpublished data)(data downloaded from: <http://apdrc.soest.hawaii.edu/las8/UI.vm>). We overlaid this trend on the existing time series of temperature [[17](#_ENREF_17)] for each Atlantis polygon to maintain spatial differences around Guam and create a time series out to 2047 (Fig A1). Bleaching occurred in the first decade in 2000 where coral somewhat recovered again as has been shown in Guam. Effects of ocean acidification were not as well manifested in the model as could be expected from literature.


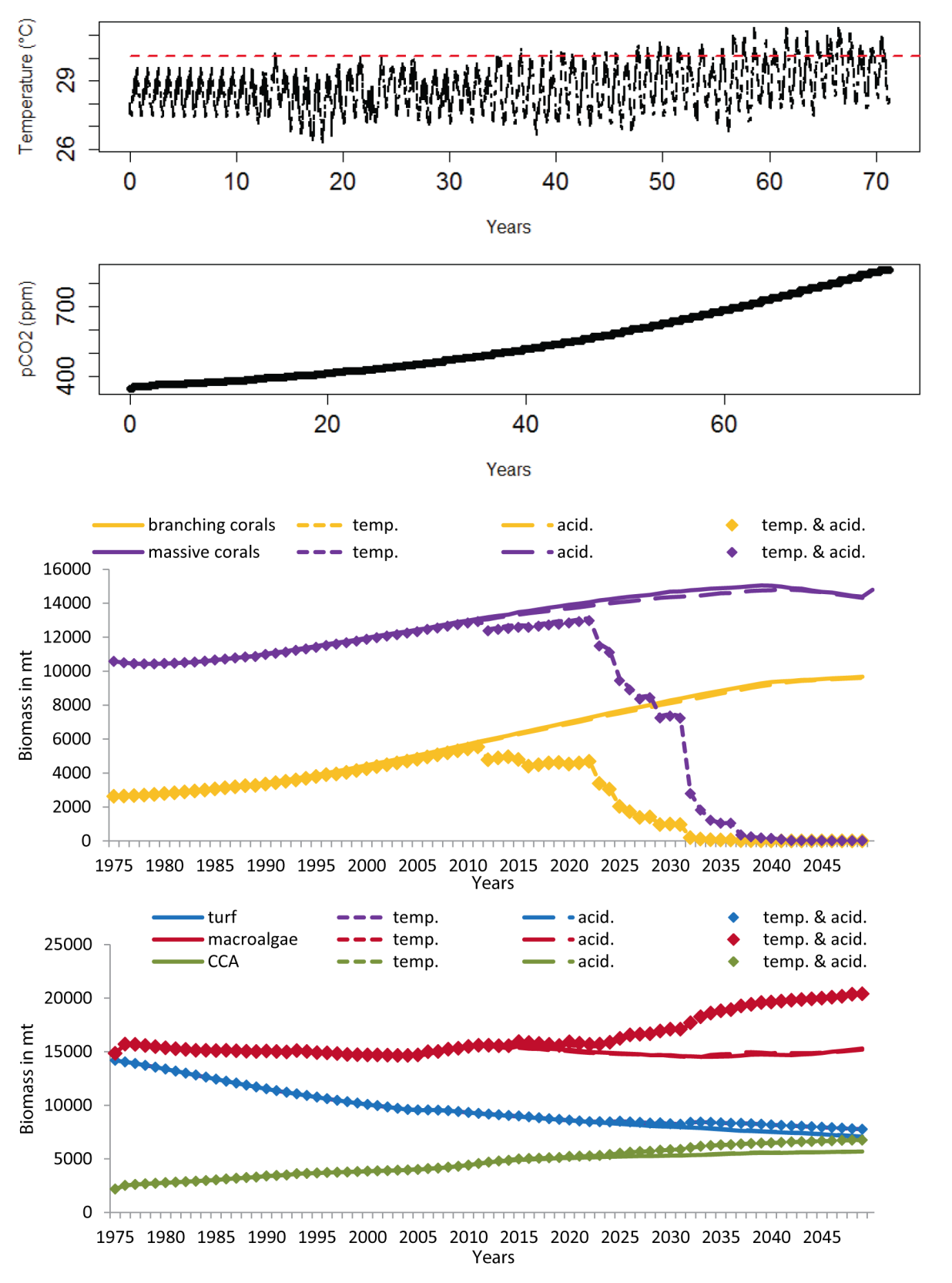


Fig A1. Trajectories of (*top*) predicted pCO_2_ (IPCC AR5 RCP8.5) and (*bottom*) sea-surface temperature (HadGEM-AO) with bleaching threshold at 30.1°C (red dashed line). Time line is from 1975–2047. These time series were used as forcing factors for future climate and ocean change. (Figure from Weijerman et al in press with permission).

We carefully validated the model in two ways following guidelines for Atlantis model development [[18-20](#_ENREF_18)]. The first validation step was to examine the model behavior over 30–75 years without any disturbances, i.e., a ‘control’ system. Biomass trajectory should stabilize at a plausible level and no functional group should go extinct. When this step was successfully completed (Fig A2) we looked at weight-at-age, which should stay stable, and abundance of size classes, which should decrease with increasing size classes. Lastly we made sure that the model was able to fit historical catch time series (Fig A3a) and that the standing stock followed the historic trend (Figs A3b).


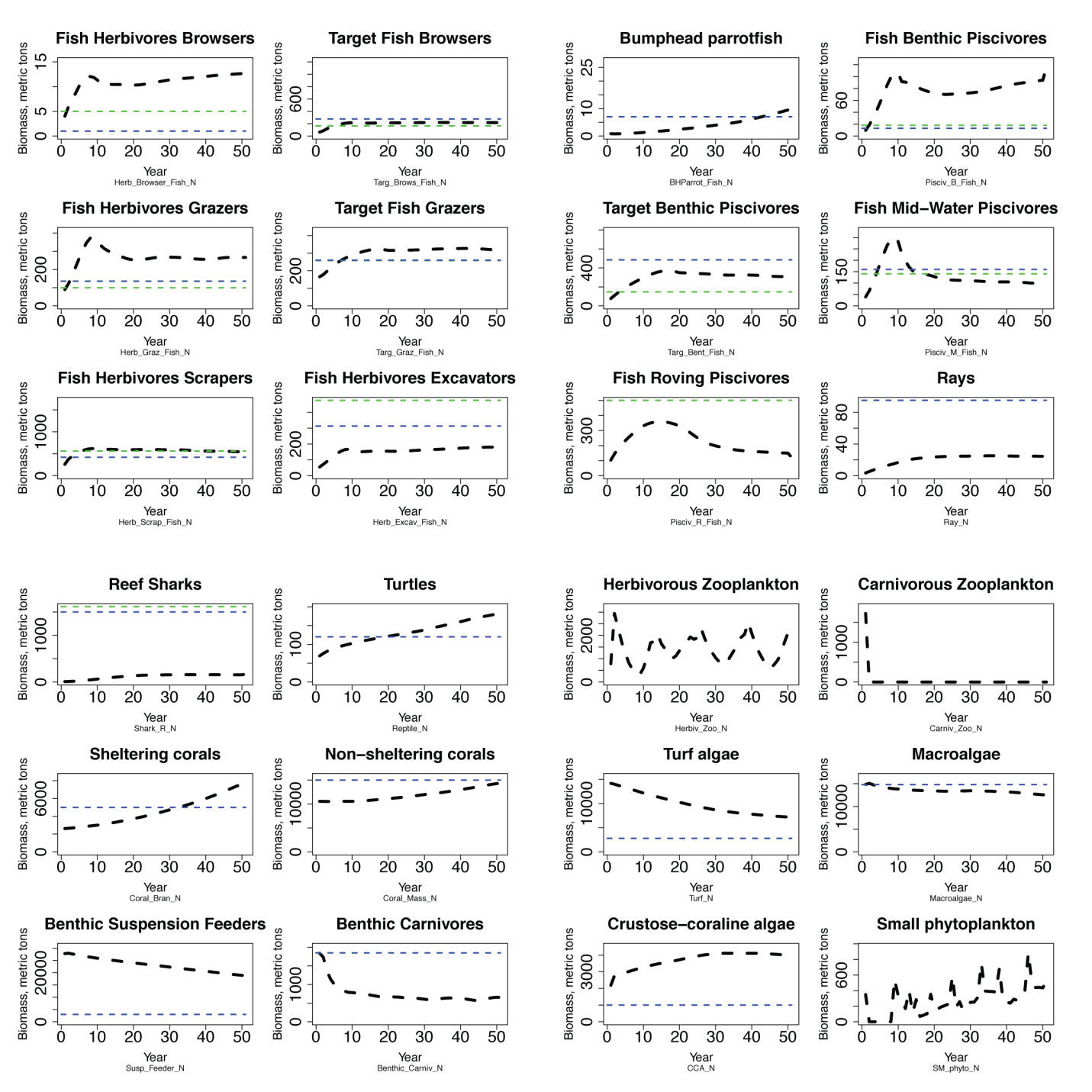


Fig A2 Biomass trajectories of a no stress simulation. (Figure from Weijerman et al in press with permission).


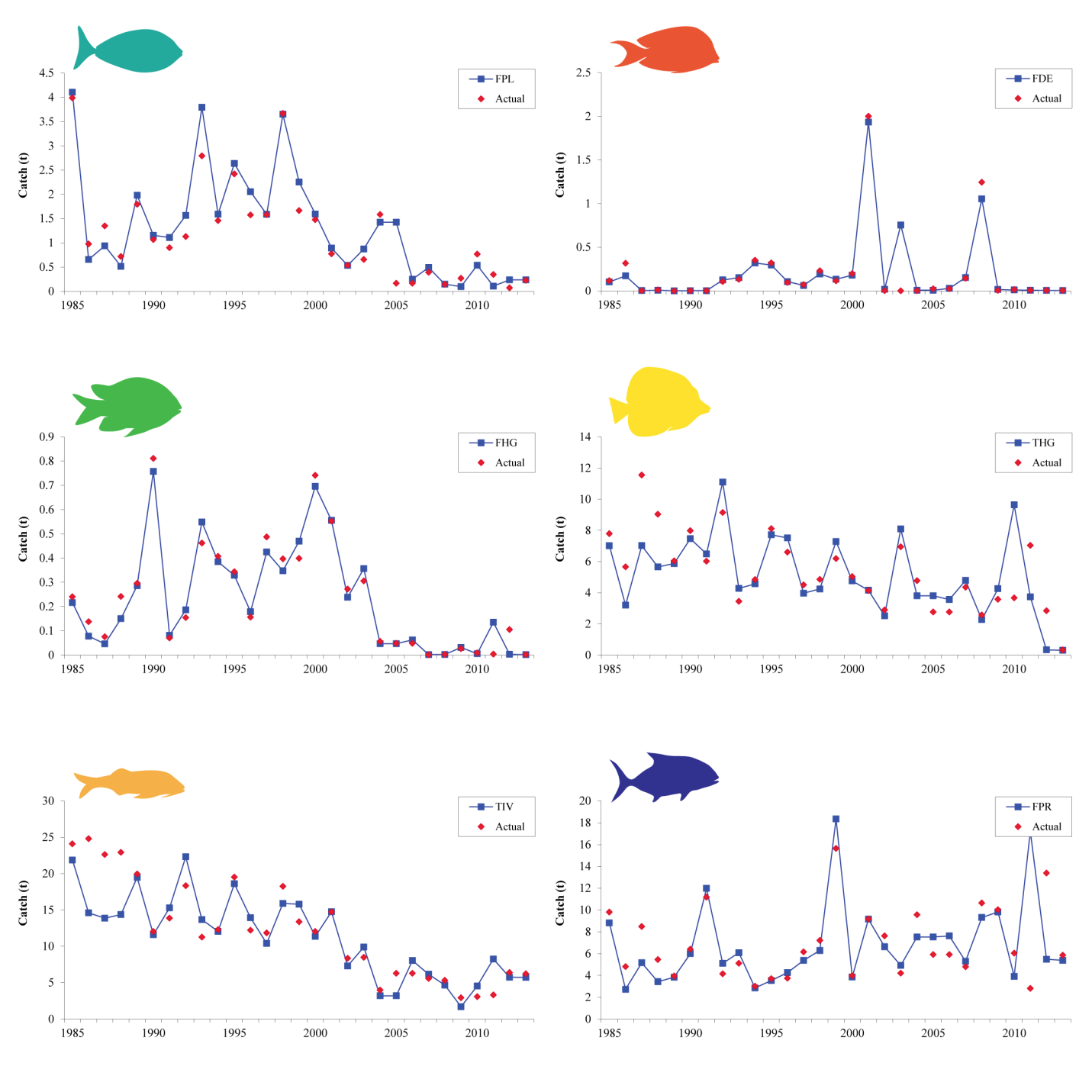


Fig A3a. Modeled (in blue line) and actual (red dots) historical catches. (Figure from Weijerman et al in press with permission).


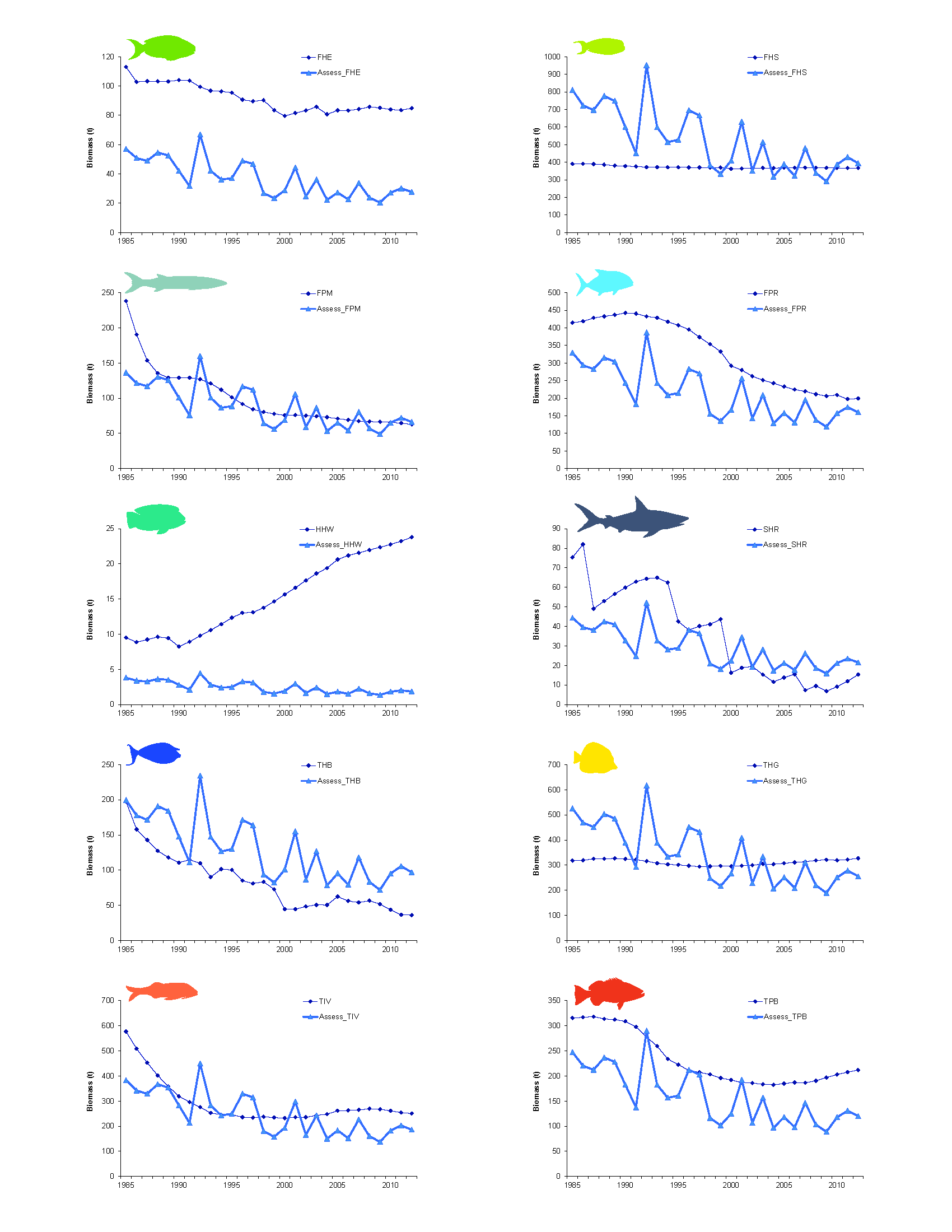


Fig A3b. Modeled (skinny dark blue line with diamonds) and historic (fat light blue line with triangles) time series of standing stock fish biomass. (Figure from Weijerman et al in press with permission).

The second validation step was to compare model projections for historical periods to available abundance time series (fish biomass and catch data) or by pattern matching [[21](#_ENREF_21)], where coral biomass trajectories from simulations of each of the main disturbances (climate change, sediments, and nutrients) were compared with results of empirical studies from particular sites in Guam or from regional sites if local information was not available (Fig A4).


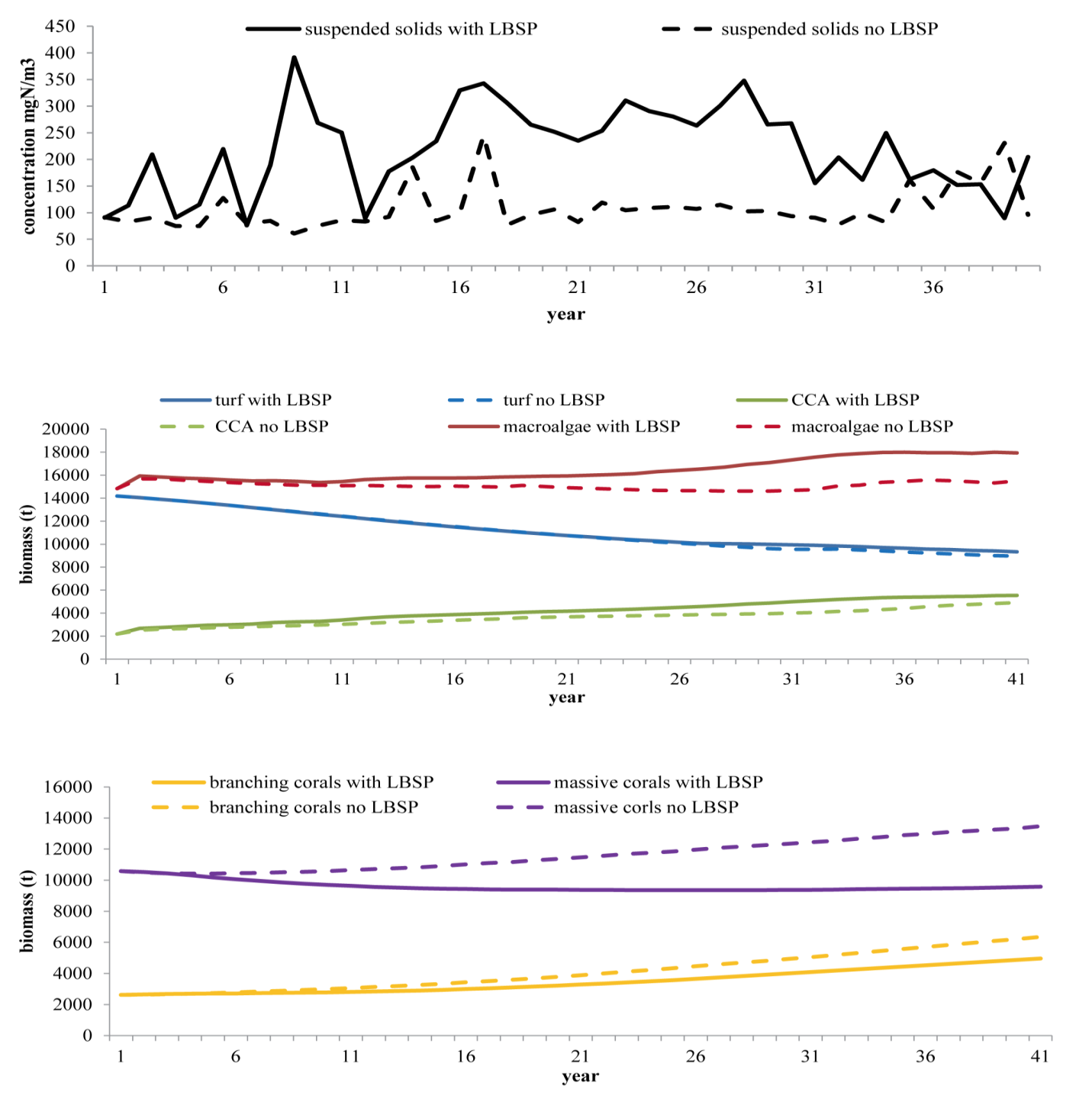
Fig A4 Biomass trajectories of a simulation additional loads of nutrients and sediments to the system. (Figure from Weijerman et al in press with permission).

Results from sensitivity analyses (primary productivity, coral-algal space competition, structural complexity) showed that uncertainty in primary productivity had the greatest influence on the model outcomes [[4](#_ENREF_4)]. Obtaining better growth rates and biomass estimates of phytoplankton communities, and improving the relationship between reef organisms and acidification and obtaining more accurate downscaled time series of projected change in pCO_2_ and will likely enhance the model’s capabilities to make projections [[4](#_ENREF_4)]. Additionally, the model skill in estimating fish biomass had a clear bias and overestimated a number of groups [[4](#_ENREF_4)]. More research is necessary to explain this bias and then correct for it (e.g., better fishery data, diet data of apex predators, recruitment data for the overestimated fish groups). However, with the current information available it is still possible to make relative comparisons [[4](#_ENREF_4)].

**References for S1 Text**

1. Fulton E, Smith A. Lessons learnt from a comparison of three ecosystem models for Port Phillip Bay, Australia. African Journal of Marine Science. 2004;26(1):219-43.

2. Fulton EA, Parslow JS, Smith AD, Johnson CR. Biogeochemical marine ecosystem models II: the effect of physiological detail on model performance. Ecological Modelling. 2004;173(4):371-406.

3. Fulton EA, Smith ADM, Johnson CR. Effects of spatial resolution on the performance and interpretation of marine ecosystem models. Ecological Modelling. 2004;176(1-2):27-42. doi: DOI: 10.1016/j.ecolmodel.2003.10.026.

4. Weijerman M, Fulton E, Kaplan I, Gorton B, Leemans R, Mooij W, et al. An integrated coral reef ecosystem model to support resource management under a changing climate. PLoS ONE. in press.

5. Weijerman M, Kaplan IC, Fulton EA, Gorton R, Grafeld S, Brainard R. Design and parameterization of a coral reef ecosystem model for Guam. U.S. Dep. Commer., NOAA Tech. Memo., NOAA-TM-NMFS-PIFSC-43, 2014.

6. Weijerman M, Williams ID, Gutierrez J, Grafeld S, Tibbats B, Davis G. Coral reef-fish biomass trends based on shore-based creel surveys in Guam. Fisheries bulletin. in press.

7. Langdon C, editor Review of experimental evidence for effects of CO_2_ on calcification of reef builders. Proceedings of the 9th International Coral Reef Symposium; 2002; Bali, Indonesia.

8. Cohen AL, Holcomb M. Why corals care about ocean acidification: uncovering the mechanism. Oceanography. 2009;22:118-27.

9. Kroeker KJ, Kordas RL, Crim RN, Singh GG. Meta-analysis reveals negative yet variable effects of ocean acidification on marine organisms. Ecology Letters. 2010;13(11):1419-34.

10. Shaw EC, McNeil BI, Tilbrook B. Impacts of ocean acidification in naturally variable coral reef flat ecosystems. Journal of Geophysical Research: Oceans. 2012;117(C3):C03038. doi: 10.1029/2011jc007655.

11. Langdon C, Atkinson MJ. Effect of elevated pCO2 on photosynthesis and calcification of corals and interactions with seasonal change in temperature/irradiance and nutrient enrichment. Journal of Geophysical Research: Oceans. 2005;110(C9):2156-202. doi: 10.1029/2004jc002576.

12. Feely RA, Doney SC, Cooley SR. Ocean acidification: present conditions and future changes in a high-CO2 world. Oceanography. 2009;22(4):36-47.

13. Anthony KRN, Kline DI, Diaz-Pulido G, Dove S, Hoegh-Guldberg O. Ocean acidification causes bleaching and productivity loss in coral reef builders. Proceedings of the National Academy of Sciences. 2008;105(45):17442-6. doi: 10.1073/pnas.0804478105. PubMed PMID: ISI:000260981800051.

14. Jokiel PL, Coles SL. Response of Hawaiian and other Indo-Pacific reef corals to elevated temperature. Coral Reefs. 1990;8(4):155-62.

15. McClanahan TR. The relationship between bleaching and mortality of common corals. Marine Biology. 2004;144(6):1239-45.

16. Donner SD, Skirving WJ, Little CM, Oppenheimer M, Hoegh-Guldberg O. Global assessment of coral bleaching and required rates of adaptation under climate change. Global Change Biology. 2005;11:2251-65. doi: 10.1111/j.1365-2486.2005.01073.x.

17. Castruccio FS, Curchitser EN, Kleypas JA. A model for quantifying oceanic transport and mesoscale variability in the Coral Triangle of the Indonesian/Philippines Archipelago. Journal of Geophysical Research: Oceans. 2013;118(11):6123-44.

18. Link JS, Fulton EJ, Gamble RJ. The northeast US application of ATLANTIS: A full system model exploring marine ecosystem dynamics in a living marine resource management context. Progress In Oceanography. 2010;87(1-4):214-34.

19. Ainsworth CH, Kaplan IC, Levin PS, Cudney-Bueno R, Fulton EA, Mangel M, et al. Atlantis model development for the Northern Gulf of California. U.S. Dept. Commer., 2011.

20. Horne P, Kaplan I, Marshall K. Design and parameterization of a spatially explicit ecosystem model of the central California Current. Technical Memo, NMFS-NWFSC-104, Dept. of Commerce, NOAA; 2010. p. 140.

21. Stow CA, Jolliff J, McGillicuddy Jr DJ, Doney SC, Allen J, Friedrichs MA, et al. Skill assessment for coupled biological/physical models of marine systems. Journal of Marine Systems. 2009;76(1):4-15.
