# Supplementary material for: Management Strategy Evaluation Applied to Coral Reef Ecosystems in Support of Ecosystem-Based Management
Source: PLoS One. 2016 Mar 29;11(3):e0152577. doi: 10.1371/journal.pone.0152577 (PMC4811577; doi:10.1371/journal.pone.0152577)
Supplement: S2 Text — (DOCX) [file pone.0152577.s008.docx]

# S2 Text. Justification of selection of performance metrics

Performance of each of the simulated scenarios was based on four management goals:

1. *Improved water quality* (no additional LBSP). The metric used to assess the performance of this goal was benthic calcifiers to non-calcifiers ratio with calcifiers defined as corals and crustose-coralline algae (CCA) and non-calcifiers as turf and macroalgae. We left out the calcifying upright macroalgal species since this metric is indicative of the space competition between corals and algae and the upright calcifying macroalgae do not compete or facilitate corals in the same way as fleshy macroalgae or CCA do, respectively. High concentrations of sediments and nutrients favor macroalgal growth over coral growth influencing the physical and ecological controls of coral-macroalgal dynamics [[1-3](#_ENREF_1)]. Additionally, terrigenous sediment smothers corals and other substrate thereby lowering coral growth and recruitment [[4](#_ENREF_4)]. Reefs in the southern part of Guam have experienced high sediment concentrations for around the last 30 years, because of coastal construction and the clearing and burning of upslope watersheds, the steep slopes wash large volumes of sediment into nearshore waters during heavy rainfall events [[5-7](#_ENREF_5)]. Over the same time period, severe coral recruitment failure has been observed [[8](#_ENREF_8)]. An important function of corals and crustose coralline algae is the three-dimensional structure they create through growth. This complexity gives refuge to numerous species and as such maintains diversity and enhances fisheries productivity [[9](#_ENREF_9), [10](#_ENREF_10)].
2. *Increased reef resilience*. Performance metrics for this goal were biomass of herbivorous fish groups split in to (a) browsers, detritivores and grazers and (b) scrapers and excavators (i.e., parrotfishes).“Reef resilience, i.e., the capability to recover after a disturbance, can be enhanced by a diverse herbivorous fish population [[11](#_ENREF_11), [12](#_ENREF_12)]. These herbivores can keep algal communities in cropped states and thereby tip the competitive balance towards corals [[13-15](#_ENREF_13)]. Large-bodied herbivores are believed to be particularly important in this regard [[16](#_ENREF_16), [17](#_ENREF_17)]. Herbivorous urchins are not abundant in Guam [[18](#_ENREF_18)] and therefore we assume are locally less important as grazers. Consequently, we focus on the various herbivorous fish species pooled into functional groups based on their complementary feeding behavior [[19](#_ENREF_19)]. The functional groups we used are: browsers (e.g. unicorn fishes, *Naso lituratus*), which reduce the biomass of upright algae; grazers and detritivores (surgeonfishes, *Acanthurus sp*), which maintain algal assemblages in cropped states; scrapers (small-bodies parrotfish, e.g. *Chlorurus sordidus*), which eliminate algal growth on coral colonies; and excavators (large-bodied parrotfish, e.g. *Chlorurus frontalis*), which open up space for coral recruitment [[14](#_ENREF_14), [20](#_ENREF_20)]. The presence of the full suite of functional guilds of herbivorous fishes can drive positive feedbacks on the benthic community composition and so aid in the recovery of reefs after disturbances [[21](#_ENREF_21)].
3. *Enhanced fish biomass*. Modeled outcome of total fish biomass was used as the metric for the performance of this goal. High total fish biomass has been rated as important to ocean users [[22](#_ENREF_22)]. Additionally, a high biomass sustains the integrity of ecosystems as it incorporates a range of desirable characteristics (e.g. large breeding stock, diverse ecological functions, relatively intact ecosystem state including all trophic levels), which in turn therefore means the ecosystem is more likely to recover from disturbances and to sustain extraction than if it was a low biomass system [[4](#_ENREF_4), [15](#_ENREF_15), [23-26](#_ENREF_23)].
4. *Maintenance of, or improved fishery landings*. Performance for this goal was measured by two fishery-related metrics: (a) the number of functional fish groups that are not overexploited, and (b) landings of reef fish. Recruitment overfishing (the reduction of a spawning stock past a point at which the stock can no longer replenish itself) can become an irreversible problem leading to fishery collapse [[27](#_ENREF_27)]. We defined a functional group overexploited when the ratio of the spawning stock biomass (SSB) to the ‘virgin’ (i.e. absence of fishing mortality) SSB was below 30% [[28](#_ENREF_28)]. Landing of reef fishes is an important social metric. In Guam, many fishers fish for cultural or social reasons instead of economic reasons and the majority of fishers share their catches which makes fishing a social activity [[29](#_ENREF_29)].

**References for S2 Text**

1. Hughes TP. Catastrophes, phase shifts, and large-scale degradation of a Caribbean coral reef. Science-AAAS-Weekly Paper Edition. 1994;265(5178):1547-51.

2. Lapointe BE. Nutrient thresholds for bottom-up control of macroalgal blooms and coral reefs. Limnol Oceanogr. 1997;44:1586-92.

3. Mumby P, Hastings A, Edwards H. Thresholds and the resilience of Caribbean coral reefs. Nature. 2007;450(7166):98-101. doi: 10.1038/nature06252.

4. Marshell A, Mumby PJ. Revisiting the functional roles of the surgeonfish Acanthurus nigrofuscus and Ctenochaetus striatus. Coral Reefs. 2012;31(4):1093-101. doi: 10.1007/s00338-012-0931-y.

5. Wolanski E, Richmond RH, Davis G, Bonito V. Water and fine sediment dynamics in transient river plumes in a small, reef-fringed bay, Guam. Estuarine, coastal and shelf science. 2003;56(5–6):1029-40. doi: 10.1016/s0272-7714(02)00321-9.

6. Wolanski E, Richmond RH, McCook L. A model of the effects of land-based, human activities on the health of coral reefs in the Great Barrier Reef and in Fouha Bay, Guam, Micronesia. Journal of Marine Systems. 2004;46(1-4):133-44. doi: DOI: 10.1016/j.jmarsys.2003.11.018.

7. Burdick D, Brown V, Asher J, Caballes M, Gawel M, Goldman L, et al. Status of coral reef ecosystems of Guam. Guam: Bureau of Statistics and Plans, Guam Coastal Management Program, 2008.

8. Minton D, Lundgren I, Pakenham A. A two-year study of coral recruitment and sedimentation in Asan Bay, Guam. Guam: National Park Service; 2007. 43 p.

9. Coker DJ, Wilson SK, Pratchett MS. Importance of live coral habitat for reef fishes. Reviews in Fish Biology and Fisheries. 2014;24(1):89-126. doi: 10.1007/s11160-013-9319-5. PubMed PMID: WOS:000331654600004.

10. Rogers A, Blanchard JL, Mumby PJ. Vulnerability of Coral Reef Fisheries to a Loss of Structural Complexity. Current Biology. 2014;24(9):1000-5.

11. Bellwood DR, Hoey AS, Hughes TP. Human activity selectively impacts the ecosystem roles of parrotfishes on coral reefs. Proceedings of the Royal Society B: Biological Sciences. 2011.

12. Heenan A, Williams ID. Monitoring herbivorous fishes as indicators of coral reef resilience in American Samoa. PLoS ONE. 2013;8(11):e79604. doi: 10.1371/journal.pone.0079604.

13. Mumby PJ, Harborne AR, Williams J, Kappel CV, Brumbaugh DR, Micheli F, et al. Trophic cascade facilitates coral recruitment in a marine reserve. Proceedings of the National Academy of Sciences. 2007;104(20):8362-7.

14. Hoey AS, Bellwood DR. Suppression of herbivory by macroalgal density: a critical feedback on coral reefs? Ecology Letters. 2011;14(3):267-73. doi: 10.1111/j.1461-0248.2010.01581.x.

15. Bejarano S, Golbuu Y, Sapolu T, Mumby PJ. Ecological risk and the exploitation of herbivorous reef fish across Micronesia. Marine Ecology Progress Series. 2013;482:197-215.

16. Lokrantz J, Nyström M, Thyresson M, Johansson C. The non-linear relationship between body size and function in parrotfishes. Coral Reefs. 2008;27(4):967-74. doi: 10.1007/s00338-008-0394-3.

17. Jayewardene D. A factorial experiment quantifying the influence of parrotfish density and size on algal reduction on Hawaiian coral reefs. Journal of Experimental Marine Biology and Ecology. 2009;375(1-2):64-9.

18. Brainard R, Asher J, Blyth-Skyrme V, Coccagna E, Dennis K, Donovan M, et al. Coral reef ecosystem monitoring report of the Mariana Archipelago: 2003-2007. Honolulu: Pacific Islands Fisheries Science Center, PIFSC Special Publication, SP-12-01; 2012. p. 1019.

19. Bellwood DR, Hughes TP, Hoey AS. Sleeping functional group drives coral-reef recovery. Current Biology. 2006;16(24):2434-9. doi: 10.1016/j.cub.2006.10.030. PubMed PMID: ISI:000243039800026.

20. Bellwood DR, Choat JH. A functional analysis of grazing in parrotfishes (family Scaridae): the ecological implications. Environmental Biology of Fishes. 1990;28(1):189-214. doi: 10.1007/bf00751035.

21. Mumby P. The impact of exploiting grazers (Scaridae) on the dynamics of Caribbean coral reefs. Ecological Applications. 2006;16(2):747-69.

22. Williams ID, Polunin NVC. Differences between protected and unprotected reefs of the western Caribbean in attributes preferred by dive tourists. Environmental Conservation. 2000;27(04):382-91. doi: doi:10.1017/S0376892900000436.

23. Jennings S, Polunin NVC. Impacts of predator depletion by fishing on the biomass and diversity of non-target reef fish communities. Coral Reefs. 1997;16(2):71-82. doi: 10.1007/s003380050061.

24. Friedlander AM, DeMartini EE. Contrasts in density, size, and biomass of reef fishes between the northwestern and the main Hawaiian islands: the effects of fishing down apex predators. Marine Ecology-Progress Series. 2002;230:253-64. PubMed PMID: ISI:000175588600022.

25. Dulvy NK, Freckleton RP, Polunin NVC. Coral reef cascades and the indirect effects of predator removal by exploitation. Ecology Letters. 2004;7(5):410-6. doi: 10.1111/j.1461-0248.2004.00593.x. PubMed PMID: ISI:000221011000008.

26. Lindfield SJ, McIlwain JL, Harvey ES. Depth refuge and the impacts of SCUBA spearfishing on coral reef fishes. PLoS ONE. 2014;9(3):e92628. doi: 10.1371/journal.pone.0092628.

27. Taylor BM, McIlwain JL, Kerr AM. Marine reserves and reproductive biomass: A case study of a heavily targeted reef fish. PLoS ONE. 2012;7(6):e39599. doi: 10.1371/journal.pone.0039599.

28. Restrepo V. Technical guidance on the use of precautionary approaches to implementing National Standard 1 of the Magnuson-Stevens Fishery Conservation and Management Act: US Department of Commerce, National Oceanic and Atmospheric Administration, National Marine Fisheries Service; 1998. 54 p.

29. Allen S, Bartram P. Guam as a fishing community. Pacific Islands Fis. Sci. Cent., NMFS, NOAA, Honolulu HI 96822-2396: Pacific Islands Fish.Sci. Cent. , 2008.
